# LncRNA Gm26917 regulates inflammatory response in macrophages by enhancing Annexin A1 ubiquitination in LPS-induced acute liver injury

**DOI:** 10.3389/fphar.2022.975250

**Published:** 2022-11-01

**Authors:** Qing Zhao, Meng-Fei Sheng, Yao-Yun Wang, Xing-Yu Wang, Wei-Yi Liu, Yuan-Yuan Zhang, Tiao-Ying Ke, Shu Chen, Gao-Zong Pang, Liang Yong, Zhan Ding, Yu-Jun Shen, Yu-Xian Shen, Wei Shao

**Affiliations:** ^1^ Department of Microbiology and Parasitology, Anhui Provincial Laboratory of Pathogen Biology, School of Basic Medical Sciences, Anhui Medical University, Hefei, Anhui, China; ^2^ Biopharmaceutical Research Institute, Anhui Medical University, Hefei, Anhui, China; ^3^ State Key Laboratory of Virology, Hubei Key Laboratory of Cell Homeostasis, College of Life Science, Wuhan University, Wuhan, Hubei, China

**Keywords:** annexin A1, acute liver injury, lipopolysaccharide, lncRNA Gm26917, macrophage, inflammation

## Abstract

Long noncoding RNAs (lncRNAs) are defined as transcripts of more than 200 nucleotides that have little or no coding potential. LncRNAs function as key regulators in diverse physiological and pathological processes. However, the roles of lncRNAs in lipopolysaccharide (LPS)-induced acute liver injury (ALI) are still elusive. In this study, we report the roles of lncRNA Gm26917 induced by LPS in modulating liver inflammation. As key components of the innate immune system, macrophages play critical roles in the initiation, progression and resolution of ALI. Our studies demonstrated that Gm26917 localized in the cytoplasm of hepatic macrophages and globally regulated the expression of inflammatory genes and the differentiation of macrophages. *In vivo* study showed that lentivirus-mediated gene silencing of Gm26917 attenuated liver inflammation and protected mice from LPS-induced ALI. Furthermore, mechanistic study showed that the 3′-truncation of Gm26917 interacted with the N-terminus of Annexin A1, a negative regulator of the NF-κB signaling pathway. We also found that Gm26917 knockdown suppressed NF-κB activity by decreasing the ubiquitination of Annexin A1 and its interaction with NEMO. In addition, expression of Gm26917 in inflammatory macrophages was regulated by the transcription factor forkhead box M1 (FOXM1). LPS treatment dramatically increased the binding of FOXM1 to the promoter region of Gm26917 in macrophages. In summary, our findings suggest that lncRNA Gm26917 silencing protects against LPS-induced liver injury by regulating the TLR4/NF-κB signaling pathway in macrophages.

## Introduction

Acute liver injury (ALI) is a devastating clinical syndrome that commonly leads to multiorgan failure and death (Manka et al., 2016). Overwhelming hepatic injury in ALI often activates systemic inflammatory response syndrome (SIRS), such as septic shock (Donnelly et al., 2016). Clinical studies suggest that the reported incidence of endotoxemia associated liver disease is as high as 75%–95% in the intensive care units (ICU) (Nolan, 2010). Many factors, including drugs, alcohol, pathogens, microorganisms and viral infection, may enter the liver and cause macrophage (Kupffer cells, etc.) activation, which eventually leads to hepatic disorder (Chen et al., 2013). Hepatic macrophages account for the largest proportion of host tissue macrophages (Dong et al., 2019). Many studies found that hepatic macrophages play critical roles in homeostasis, progression and liver injury resolution (Tacke and Zimmermann, 2014; Shan and Ju, 2020; Wen et al., 2021).

Lipopolysaccharide (LPS) is the major component of endotoxin in gram-negative bacteria and causes uncontrolled production of inflammatory mediators and oxidative stress (Maldonado et al., 2016). LPS-induced liver injury in mice is a commonly employed model for ALI research, simulating the course of liver damage and failure in septic endotoxemia (Freudenberg and Galanos, 1988; Xiong et al., 1999). Following LPS-induced ALI, the number of inflammatory cells increases remarkably, especially macrophages (van der Heide et al., 2019). LPS determines the overall immune activation and response of the host through Toll-like receptor 4 (TLR4) (Matsuura, 2013; Ramachandran, 2014; Allen and Imperiali, 2019). Upon LPS stimulation, TLR4 can recognize LPS and initiate downstream signals through two main transduction signal branches, namely, myeloid differentiation factor 88 (MyD88) or Toll-interleukin-1 (IL-1) receptor (TIR) domain-containing adaptor-inducing IFNβ (TRIF) (Chattopadhyay et al., 2015; Wang et al., 2015), resulting in the activation of NF-κB and release of proinflammatory cytokines and chemokines (Han and Ulevitch, 2005; Kagan and Medzhitov, 2006; Li M. et al., 2018). Therefore, inactivation of the TLR4/NF-κB signaling pathway can attenuate LPS-induced ALI.

Annexin A1 (ANXA1) contains 346 residues and consists of two different regions, the singular N-terminal domain, also called the tail, and the C-terminal domain, named the core domain. ANXA1 is regulated by glucocorticoid and is a pivotal regulator of the innate and adaptive immune systems (D'Acquisto et al., 2008). ANXA1 has been extensively studied for its anti-inflammatory activity, but it also exhibits pro-inflammatory effects in a variety of inflammatory experimental models (Yang et al., 1999; D'Acquisto et al., 2008; Shao et al., 2019). Moreover, ANXA1 interacts with nuclear factor kappa B kinase subunit gamma (NEMO) and regulates NF-κB activity in breast cancer. Interaction between ANXA1 and NEMO was found to constitutively activate NF-κB signaling pathway (Bist et al., 2011). NEMO is a component of the IKK complex that plays a critical role in the activation of the NF-κB pathway (Shifera, 2010). The IKK complex, which consists of the catalytic subunits IKKα/IKKβ and the regulatory subunit NEMO, phosphorylates the inhibitory molecule IκB, resulting in its degradation and the translocation of NF-κB transcription factors to the nucleus. Without NEMO, the IKK complex could not be activated and the NF-κB signaling pathway was blocked (Legarda-Addison et al., 2009).

LncRNAs are defined as transcripts of more than 200 nucleotides with diverse biological functions (Guttman et al., 2009). The dysregulation of lncRNAs has also been demonstrated to lead to the progression liver injury (Zhao et al., 2017). Notably, the critical roles of lncRNAs in regulating liver diseases have been reported (Xie et al., 2019). For instance, the liver-enriched lncRNA Lfar1 could promote hepatic fibrosis by inducing HSC activation and hepatocyte apoptosis, playing a key role in controlling the activation and pyroptosis of macrophages (Zhang et al., 2020). LncRNA-H19 exosomes secreted by bile duct cells play a key role in promoting macrophage activation and liver inflammation in cholestatic liver disease (Li X. et al., 2020). To date, there have been many reports of lncRNAs involved in liver diseases. However, the roles of lncRNA in ALI are still elusive.

Gm26917 is a poorly studied lncRNA. Although, expression changes in Gm26917 have been identified in some immune-mediated diseases by transcriptome analysis, the detailed role of Gm26917 is still unknown. For example, Hewitson et al. identified the downregulation of Gm26917 in Th1-activated CD4^+^ T cells compared with naive cells by using bulk RNA-seq (Hewitson et al., 2020). Gm26917 was also found to be enriched in NLRP3 inflammasomes by high-throughput sequencing (Zhang et al., 2019). The expression of Gm26917 in T and B cells was upregulated following ultrasound treatment in arthritic mice (Zachs et al., 2019). Another study found that Forkhead box M1 (FOXM1) could bind to the promoter region of Gm26917 and directly regulate the transcription of Gm26917 in muscle satellite cells. (Chen et al., 2018).

In this study, we investigated the function of lncRNA Gm26917 in LPS-induced ALI. Significantly elevated expression of Gm26917 in LPS-induced ALI was identified by RNA sequencing and confirmed by qRT–PCR. Lentivirus-mediated Gm26917 knockdown protected mice from acute liver injury induced by LPS. Silencing of Gm26917 dramatically decreased levels of proinflammatory factors globally and induced macrophage M2 polarization. Furthermore, Gm26917 acted as a positive regulator of inflammatory responses by interacting with ANXA1 and promoting its ubiquitination. The expression of G26917 in LPS-induced inflammatory macrophages was found to be regulated by FOXM1. Collectively, Our study showed for the first time a detailed role of Gm26917 in macrophage inflammatory responses.

## Materials and methods

### Experimental animals

C57BL/6 mice (6–8 weeks old) were purchased from the Experimental Animal Center of Anhui Medical University and maintained in an SPF animal room. All experiments and animal care were approved by the Anhui Medical University Institutional Animal Care and Use Committee and Ethics Committee. Lentiviruses (LV-sh-NC or LV-sh-Gm26917, 1 × 109 TU/ml) were delivered into mice by tail vein injection 3 days before establishing the LPS-induced ALI model. For LPS-induced liver injury, mice were injected intraperitoneally with LPS (*Escherichia coli* O111: B4, Sigma–Aldrich, St. Louis, MO) at a dose of 2.5 mg/kg (Possamai et al., 2010). Control animals were administered equivalent volumes of PBS. All mice were sacrificed 2 h after LPS injection.

### Primary mouse hepatocyte and hepatic nonparenchymal cell isolation

Primary hepatocytes and hepatic nonparenchymal cells were isolated from 6- to 8-week-old C57BL/6 mice by a 2-step collagenase type IV perfusion method (Yang et al., 2019). In brief, mice were sacrificed before blood samples were obtained after enucleating the eyeballs. The liver was removed and washed with ice-cold PBS. Then, liver samples were minced, and cells were dispersed by treatment with 0.2% collagenase type IV (Sigma) at 37°C for 30 min with a Laboratory Shaker at a speed of 250 rpm. The cells were then filtered through a 200-mesh screen into a new tube. After centrifugation (5 min at 50 g), primary hepatocytes in the cell pellet were collected. For purification of the hepatic nonparenchymal cells, the supernatant was collected by centrifugation at 2,500 rpm for 5 min. Pellets were resuspended in 40% Percoll, and the gradient was centrifuged at 2,500 rpm for 30 min. ACK lysis solution were added to the lower layer and placed on ice for 5 min to remove red blood cells.

### Isolation of peritoneal macrophages

Peritoneal macrophages were isolated from C57BL/6 mice. In brief, the abdominal cavity was injected with 5 ml ice-cold PBS. Lavage fluid was collected and centrifuged. Peritoneal exudate cells were resuspended in complete RPMI 1640 and plated in tissue culture dishes. Cells were incubated for 2 h, and then nonadherent cells were removed by washing with PBS.

### RNA-seq analyses

Total RNA was extracted from livers of mice stimulated with LPS for 2 h and three livers of control mice. cDNA libraries were constructed and sequenced on an Illumina HiSeq platform, and 150-bp paired-end reads were generated. Raw reads were first processed to obtain clean reads. Clean reads were mapped to the mouse genome (mm10 version) by STAR v2.6.1a (https://github.com/alexdobin/STAR), and then the Counts v1.6.3 (http://bioinf.wehi.edu.au/featureCounts/) and stringtie v2.0 (https://ccb.jhu.edu/software/stringtie/) were used to count the read numbers mapped to each gene and calculate the FPKM per gene, respectively. Differential expression analysis of each group was performed using DESeq2 v1.22.2 (https://bioconductor.org/packages/release/bioc/html/DESeq2.html), and genes with corrected *p* values < 0.05 and |log2FC|>1 were assigned as significantly differentially expressed genes (DEGs). GO/KEGG enrichment of DEGs was implemented using the KAAS - KEGG Automatic Annotation Server (genome.jp) and GO/KEGG terms with a corrected *p*-value<0.05 were considered significantly enriched.

### RNA extraction, RT–PCR and qRT–PCR

Total RNA was obtained using TRIzol reagent (Invitrogen, 15596-018). cDNA was synthesized using a cDNA synthesis kit (11706, Accurate Biology) following the manufacturer’s instructions. Relative gene expression was determined by quantitative real-time PCR using the SYBR Green PCR Master Mix kit (11701, Accurate Biology) and performed using the CFX96 Real-Time PCR System (CFX96, Bio-Rad, USA). The expression value was calculated using the comparative Ct method with the Formula ^2−ΔCt^, and the expression levels of each gene was normalized to Gapdh. The primer sequences are listed in Supplementary Table S1.

### Histological analysis of liver

Tissues were removed from mice, and paraffin sections were prepared and stained with hematoxylin and eosin. The degree of inflammation and injury on the liver was graded semiquantitatively from 0 to 4: inflammation score 0 = no evidence for inflammation; 1 = low level of inflammation with scattered infiltrating inflammatory cells (1–2 foci only); 2 = moderate inflammation with multiple foci; 3 = high levels of inflammation with increased hepatocyte edema and severe inflammatory cell infiltration; 4 = maximal severity of inflammation with exacerbated liver inflammation, hemorrhaging, and sinusoidal dilatation. The results of HE are evaluated by the size of the area infiltrated by inflammatory cells and the average number of inflammatory cells under the high-power field of view, and statistical data are made into histograms for statistical comparison. Image pro-plus (IPP) software is used to calculate the size of the area infiltrated by inflammatory cells. 10 samples were analyzed for each group.

### RNA fluorescence *in situ* hybridization and protein immunofluorescence

The Cy3-labeled lncRNA Gm26917 probe was designed and synthesized by GenePharma (Shanghai, China). The probe sequences are listed in Supplementary Table S1. FISH was conducted according to the manufacturer’s instructions (GenePharma). Cells grown on Collagen I 22-mm round coverslips (354089; Corning) were fixed in 4% paraformaldehyde for 15 min and treated with 0.1% Triton X-100. Then, the samples were rinsed with PBS three times and incubated with prehybridization buffer (2× saline sodium citrate, 10% formamide) at 37°C in an incubator. The FISH probes against Gm26917 were resuspended in hybridization buffer (2× saline sodium citrate, 10% formamide, 10% dextran sulfate) to a final concentration of 100 nM per probe set. Hybridization was carried out in a humidified chamber at 37°C for 12–16 h. For combined RNA FISH immunostaining, after incubation with the Gm26917 probe, samples were washed three times with PBS and treated with ANXA1 (sc-12740) or CD68 (ab125212) antibody at 37°C; 2 h later, the cells were washed three times with PBS and treated with fluorescently labeled secondary antibody (ab150080) for 1 h at 37°C. The images were obtained using a ZEISS 800 laser scanning confocal microscope (ZEISS, Jena, Germany).

### Flow cytometric analysis

The percentages of macrophages with the M1 or M2 phenotype were analysed by flow cytometry. Briefly, cultured peritoneal macrophages with Gm26917 knockdown or LPS treatment were harvested, blocked using Fc receptor blocker, and then incubated with specific antibodies for 30 min on ice. Macrophages were identified as F4/80-positive cells. The percentages of inflammatory M1 macrophages and regulatory M2 macrophages were then evaluated using antibodies against the M1 macrophage marker Ly6C (BD Pharmingen) and M2 macrophage marker CD206 (BD Pharmingen), respectively. All samples were acquired and analyzed using a CytoFLEX flow cytometer (Beckman Coulter) with CytExpert software (version 2.4).

### RNA immunoprecipitation

Cells were treated with final concentration of 0.3% formaldehyde for 10 min at 37°C, and the reaction was then stopped by adding glycine to a final concentration of 125 mM at room temperature. Cells were then washed twice with PBS and centrifuged. Cell pellets were resuspended in RIPA buffer (50 mM Tris-HCl, pH 7.5, 150 mM NaCl, 1 mM EDTA, 0.1% SDS, 0.5% Nonidet P-40, 1.0% Triton X-100, 0.5 mM DTT) and vortexed on ice for 30 min. Extracts were then precleared with protein G-Sepharose beads (Beyotime). After centrifugation, whole cell lysates were incubated with ANXA1 antibodies or IgG overnight at 4°C for 4 h and then incubated with Protein G beads overnight. The pellets were washed 3 times with RIPA buffer and resuspended in 0.5 ml TRIzol reagent for subsequent qRT–PCR.

### Plasmids and antibodies

The pcDNA-His-ANXA1 and two truncated constructs of ANXA1 (1-185 aa, 186-346 aa) were all created by cloning them into the pcDNA3.1 vector with the CMV promoter and the appropriate epitope tags. HA-Ub-WT, HA-Ub-K6, HA-Ub-K11, HA-Ub-K27, HA-Ub-K29, HA-Ub-K33, HA-Ub-K48 and HA-Ub-K63 plasmids were kind gifts from Professor Ronggui Hu (Chinese Academy of Sciences). To construct lncRNA Gm26917 overexpression plasmids, Gm26917 cDNA was synthesized and cloned into the pCDH vector by Miaoling (Wuhan, China).

Antibodies used for Western blotting, coimmunoprecipitation, or immunofluorescence were as follows: ANXA1 (EH17a, sc-12740, Santa Cruz), GAPDH (T004, Affinity), anti-HA antibody (Y-11, sc-805, Santa Cruz), His antibody (ZENBIO, 230001), NEMO (F-10, sc-166398, Santa Cruz), p65 (ZENBIO, 250021), p-p65 (ZENBIO, 310013), IKK-α (C-6, sc-166231, Santa Cruz), p-IKK-α/β (CST, #2694, USA), F4/80 (C-7, 377009, Santa Cruz), CD68 (ab125212, Abcam), FOXM1 (EPR17379, ab207298, Abcam).

### Luciferase assay

The NF-κB luciferase reporter assay was performed as previously described (Chen et al., 2015). Cells were cotransfected with siRNA and reporters as indicated, followed by stimulation with LPS. Then, the cells were harvested and lysed, and luciferase activity was determined using a dual-luciferase reporter assay system (Promega, WI, USA) according to the manufacturer’s instructions. Values obtained from firefly luciferase signals were normalized to Renilla luciferase activity.

### SiRNA and recombinant lentivirus

The siRNA and recombinant lentivirus were designed and produced by GenePharma Co., Ltd. (Shanghai, China). To generate lentivirus expressing siRNA against Gm26917 (LV-sh-Gm26917), 3 siRNAs for mouse Gm26917 were designed, and the one with the optimal knockdown efficiency (sequence: 5′-CAA​AAC​CAA​CCC​GGT​GAG​C-3′) was chosen to create LV-sh-Gm26917. Amplification and purification of recombinant lentivirus was performed according to the manufacturer’s instructions (GenePharma Co., Ltd., Shanghai, China).

### Cytometric bead array

Cytokine levels were analyzed using a cytometric bead array. Supernatants were collected, and cytometric bead array assays were performed using the Murine Inflammation Cytometric Bead Array kit according to the manufacturer’s instructions. Data were collected on a FACSC alibur using CellQuest software and analyzed using BD Cytometric Bead Array software (BD Biosciences).

### Immunohistochemistry and histopathology

Tissues were fixed with 4% paraformaldehyde and embedded in paraffin. The embedded tissue was cut into 4-μm-thick serial sections. The sections were deparaffinized in xylene, hydrated through graded ethanol and stained with hematoxylin and eosin (HE).

For immunohistochemistry staining, the slides were deparaffinized, and antigen retrieval was performed. The slides were then blocked with goat serum and incubated with antibodies. Subsequently, the sections were stained with 3,3′-diaminobenzidine tetrahydrochloride (DAB) and hematoxylin. The images were obtained with a microscope and analyzed with image-processing software (ImageJ v 1.48).

### Alanine aminotransferase and aspartate aminotransferase measurements

The serum ALT and AST concentrations were assessed using the AST/GOT Assay Kit and ALT/GPT Assay Kit (Nanjing Jiancheng Bioengineering Institute, China) following the manufacturer’s directions.

### Chromatin immunoprecipitation

Chromatin immunoprecipitation (ChIP) was performed as described previously (Shao et al., 2016). RAW264.7 cells were treated with PBS or LPS (25 ng/ml) for 6 h, cross-linked with 1% formaldehyde at room temperature for 10 min and quenched by addition of glycine to 125 mM. After centrifugation, cell pellets were washed with cold PBS and lysed in lysis buffer (50 mM HEPES-KOH at pH 7.5, 150 mM NaCl, 1 mM EDTA, 1% Triton X-100, 0.1% Na deoxycholate, 0.1% SDS, 1 mM PMSF) containing protease inhibitors.

Chromatin was sheared using a Diagenode Bioruptor. Immunoprecipitation was performed using anti-IgG antibody (bsm-33179M-HRP), anti-FOXM1 antibody (ab207298) and ChIP-Grade Protein G Agarose Beads (Cell Signaling Technology). Associated DNA was then purified using a PCR clean-up kit (Axygen) and analyzed by QuantStudio Real-Time PCR (Thermo Fisher). PCR signals from the immunoprecipitated samples were normalized to the input and are presented as the fold change compared with the IgG control. Primer sequences are listed in Supplementary Table S1.

### Statistical analysis

GraphPad Prism 8.0 software (GraphPad Prism) was used to analyze the data. Quantification of Western blots was performed using ImageJ software. Experimental values are expressed as the means ± SEM of at least three independent experiments. The student’s t test was used to test the statistical significance of the differences between two groups. For multiple groups, significance was evaluated by one-way ANOVA. *p* < 0.05 was considered statistically significant.

## Results

### Identification of differentially expressed lncRNAs in the liver of LPS-treated mice

**FIGURE 1:**
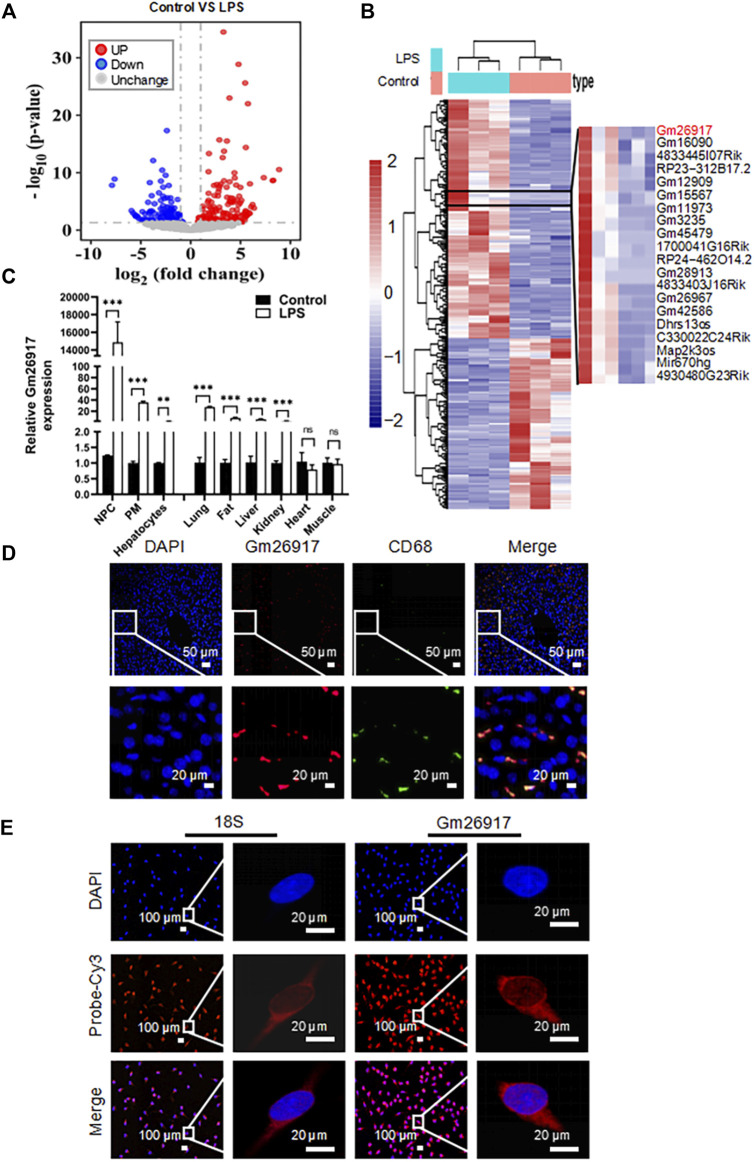
Gm26917 expression is induced in mice with LPS-induced acute liver injury. **(A)** Mice were intraperitoneally injected with LPS (2.5 mg/kg) for 2 h to establish an acute liver injury model. LncRNA profiles of the livers were analyzed by RNA sequencing. Volcano plot showing differential lncRNA expression between livers from control and LPS-treated mice. Red and blue points correspond to 2.0-fold changes in the two groups and indicate a FDR <0.05. **(B)** Heatmap of differentially expressed lncRNAs in livers from control and LPS-treated mice. **(C)** Expression of Gm26917 in various cell types and tissues in control and LPS-treated mice. **(D)** For colocalization analysis, livers were costained with Cy3-labeled anti-Gm26917 probe (red) and anti-CD68 (macrophage marker, green). Nuclei were stained with 4′,6-diamidino-2-phenylindole (DAPI) (blue). **(E)** Macrophages were stained with Cy3-labeled anti-Gm26917 probe (red) for fluorescence in situ hybridization (FISH) and counterstained with DAPI. Cy3-labeled anti-18S rRNA staining served as a positive control. Data represent the mean ± SEM of three independent experiments. ^*^ *p* < 0.05, ^**^ *p* < 0.01, ^***^ *p* < 0.001.

LPS-induced liver injury is a well-established model of acute liver failure in mice. To investigate the roles of lncRNAs in LPS-induced liver injury, the transcriptomes of three LPS-treated and three control mouse livers were sequenced using high-throughput RNA sequencing. Bioinformatics analysis showed that 167 lncRNAs were significantly upregulated (red plot) and 119 lncRNAs were downregulated (blue plot) (Figure 1A). Among them, Gm26917 was identified to be highly expressed and significantly induced in LPS-induced liver injury (Figure 1B and Supplementary Table S2).

### Gm26917 is induced in liver macrophages after LPS treatment

LPS is a potent activator of monocytes and macrophages. The liver has the largest population of resident macrophages in the body. To explore the function of Gm26917, we examined the expression of Gm26917 in different liver cells, peritoneal macrophages and tissues. Enhanced expression of Gm26917 was found in different cell types and tissues after LPS treatment. Differences in Gm26917 expression were most significant in liver non-parenchymal cells (NPCs) and peritoneal macrophages (PMs). We also detected the expression of Gm26917 in the lung, fat, liver, kidney, and intestine, but not in the heart or muscle (Figure 1C), which may have resulted from the large number of macrophages in the lung, fat, liver and kidney.

To confirm the expression of Gm26917 in liver macrophages, fluorescence *in situ* hybridization (FISH) and CD68 immunofluorescence costaining were performed using the livers of LPS-treated mice. As shown in Figure 1D, we found that CD68-positive (green) macrophages colocalized with Gm26917 (red), suggesting that Gm26917 was mainly distributed in macrophages in the liver (see also Supplementary Figures S1A,B). FISH also showed that Gm26917 was mainly located in the cytoplasm of peritoneal macrophages activated by LPS (Figure 1E), suggesting that Gm26917 might play its biological role in the cytoplasm. Taken together, LPS-induced Gm26917 may function in LPS-induced liver injury by modulating the inflammatory response in macrophages.

### Gm26917 silencing protects against LPS-induced acute liver injury

**FIGURE 2:**
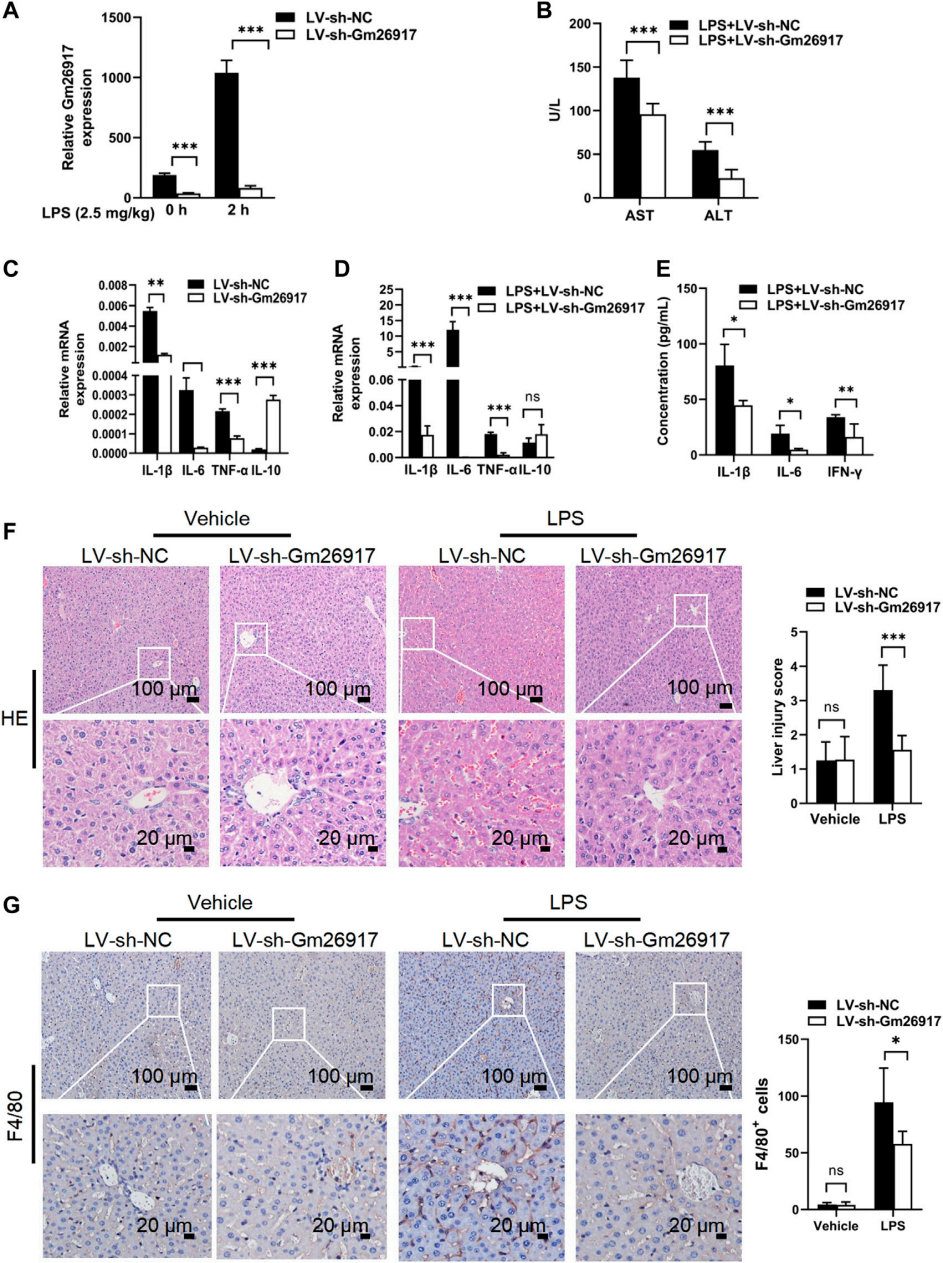
Gm26917 silencing attenuates LPS-induced liver injury. **(A)** Gm26917 was efficiently knocked down by lentivirus sh-Gm26917 in mouse livers. After 72 h of lentivirus sh-Gm26917 administration, livers were collected, expression of Gm26917 was measured by qRT–PCR. **(B)** Gm26917 silencing reduced plasma ALT and AST levels after LPS treatment. Serum ALT and AST levels in LPS-sh-NC-treated mice and LPS-sh-Gm26917-treated mice were measured. **(C,D)** Silencing of Gm26917 decreased the expression of inflammatory cytokines. **(C)** Expression levels of IL-1β, IL-6, TNF-α and IL-10 in the livers of mice infected with lentivirus for 72 h were quantified by qRT–PCR. **(D)** Expression levels of cytokines IL-1-β, IL-6, TNF-α and IL-10 in the livers of mice after 72 h of lentivirus infection followed by LPS stimulation for 2 h were quantified by qRT–PCR. **(E)** Silencing of Gm26917 reduced plasma levels of inflammatory cytokines. Mouse serum was collected, and cytokines (IL-1β, IL-6 and IFN-γ) were quantified using a cytometric bead array. **(F)** Gm26917 silencing attenuated LPS-induced liver injury in LPS-induced ALI mice. Quantitative analysis of the liver injury score. **(G)** Gm26917 silencing attenuated LPS-induced hepatic macrophage number in LPS-induced ALI mice. Quantitative analysis of F4/80-positive cells. Data represent the mean ± SEM of three independent experiments. ^*^ *p* < 0.05, ^**^ *p* < 0.01, ^***^ *p* < 0.001.

To investigate the effects of Gm26917 in mice with LPS-induced ALI, mice were first injected with LV-sh-Gm26917 *via* the tail vein. Then, mice received a single intraperitoneal injection of 2.5 mg/kg LPS after 72 h. Mice were sacrificed 2 h after LPS injection. We observed a dramatic decrease in Gm26917 in the liver before and after LPS treatment (Figure 2A). The serum samples were also analyzed for the levels of Alanine aminotransferase (ALT) and Aspartate aminotransferase (AST) liver enzymes. Elevated levels of ALT and AST in the blood signify liver damage. Compared with the animals treated with LV-sh-NC, elevated serum ALT and AST levels were ameliorated in LV-sh-Gm26917-infected mice (Figure 2B). Moreover, Gm26917 knockdown resulted in a reduction in the proinflammatory cytokines IL-1β, IL-6, and TNF-α and an increase in the anti-inflammatory cytokine IL-10 before and after LPS administration in the liver (Figures 2C,D). Additionally, mouse serum from the above experiments was collected, and the concentrations of IL-1β, IL-6 and IFN-γ were quantified using a cytometric bead array. As expected, Gm26917 knockdown reduced robust inflammatory cytokines (IL-1β, IL-6 and IFN-γ) production (Figure 2E). Moreover, examination of the pathology of the liver tissue showed that the LPS-treated mice developed exacerbated liver inflammation, hemorrhaging, and sinusoidal dilatation, whereas fewer liver lesions lower and lower liver injury scores were observed in the mice that received LV-sh-Gm26917 (Figure 2F). Additionally, the LV-sh-Gm26917 group showed decreased macrophage number (Figure 2G). In summary, Gm26917 silencing effectively relieved LPS-induced acute liver injury.

Given the important roles of Gm26917 in regulating the inflammatory processes in mice with ALI induced by LPS, we then investigated whether Gm26917 was involved in other inflammatory liver diseases. Interestingly, Gm26917 levels were elevated in alcoholic fatty liver (Supplementary Figure S1C), and Schistosoma japonicum infection caused liver fibrosis (Supplementary Figure S1D). In the liver pathology of schistosomiasis, Gm26917 was also found to colocalize predominantly with CD68-positive macrophages (Supplementary Figure S1E). Taken together, these results indicate that Gm26917 is involved in inflammatory liver diseases by regulating macrophage responses.

**FIGURE 3:**
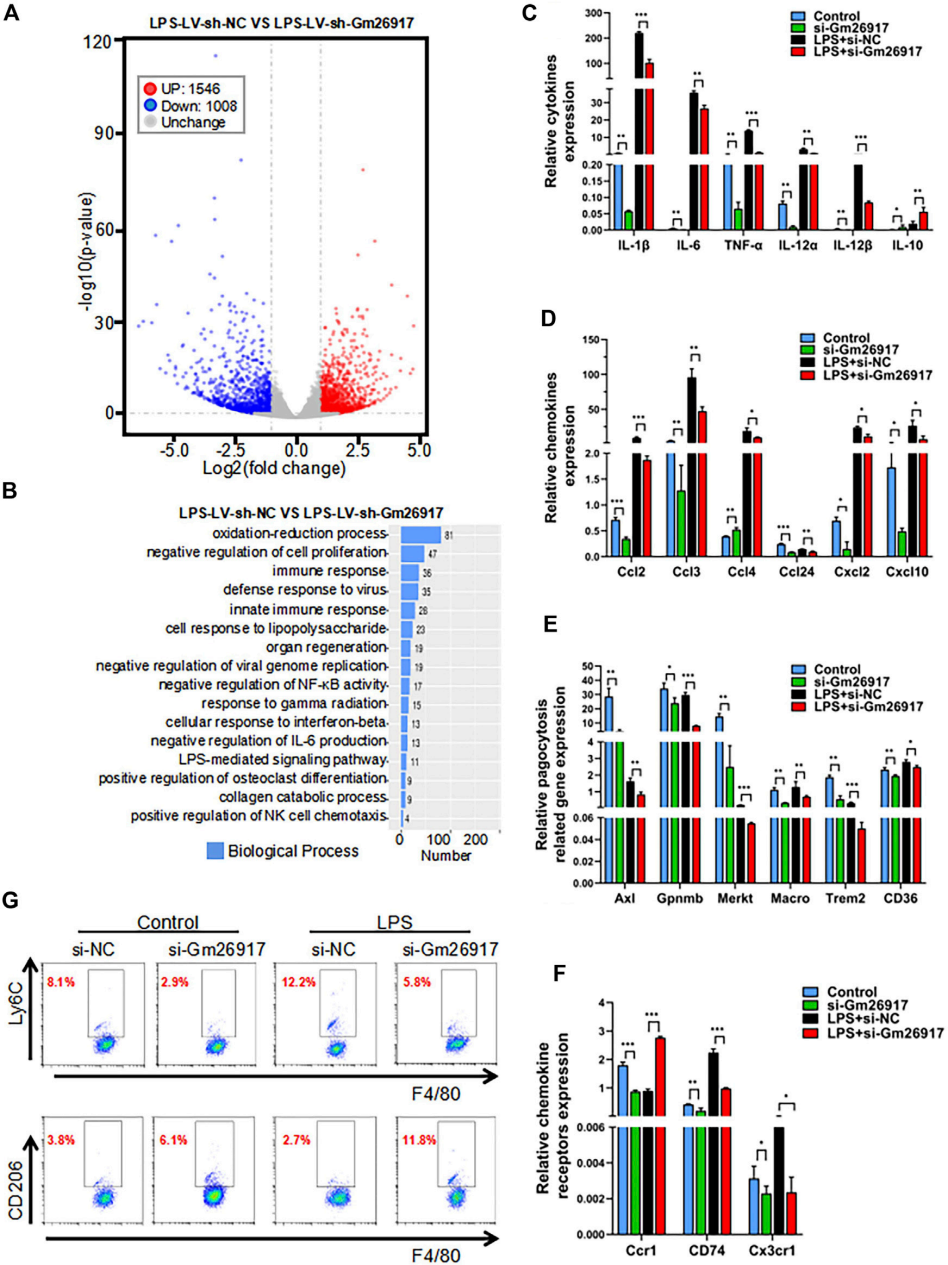
Gm26917 silencing inhibits LPS-induced macrophage-mediated inflammation. **(A)** Differential gene expression data are presented as volcano plots between sh-NC-treated cells and shRNA-Gm26917-treated peritoneal macrophages with three repeats. Red and blue points correspond to 2.0-fold changes in the two groups and indicate a FDR <0.05. **(B)** Gene ontology analysis for all genes with altered expression. **(C)** Effects of Gm26917 knockdown on the expression of inflammatory cytokines before and after LPS treatment in cultured peritoneal macrophages. **(D)** Effect of Gm26917 knockdown on the expression of chemokines before and after LPS treatment in cultured peritoneal macrophages. **(E)** Effect of Gm26917 knockdown on the expression of phagocytosis-related genes before and after LPS treatment in cultured peritoneal macrophages. **(F)** Effect of Gm26917 knockdown on the expression of chemokine receptors before and after LPS treatment in cultured peritoneal macrophages. **(G)** Flow cytometry analysis of the macrophage differentiation before and after Gm26917 knockdown under vehicle control or LPS treatment. Cells positive for the F4/80 marker were defined as macrophages, which were further sorted using the proinflammatory macrophage (M1 macrophage) marker Ly6c and the alternatively activated macrophage (M2 macrophage) marker CD206. Data represent the mean ± SEM of three independent experiments. ^*^ *p* < 0.05, ^**^ *p* < 0.01, ^***^ *p* < 0.001.

To globally analyze the roles of Gm26917 in macrophages, RNA transcriptome sequencing was performed in LV-sh-NC- or LV-sh-Gm26917-infected peritoneal macrophages treated with LPS for 6 h, Gm26917 was significantly knocked down (Supplementary Figure S2A). Different expression patterns were observed between control and Gm26917 knockdown macrophages, with increases in 1,546 genes and decreases in 1,088 genes (Figure 3A, Supplementary Figure S2B and Supplementary Table S3). Additionally, a thorough gene ontology analysis highlighted that Gm26917 silencing affected processes such as oxidative stress, regulation of cell proliferation and the immune response (Figure 3B). Analysis of the KEGG database revealed differences between the control group and Gm26917 silencing group in 30 pathways, including pathways involved in inflammation (Supplementary Figure S2C). In sum, these results showed that Gm26917 could be an important regulator of macrophage inflammatory responses.

To investigate the detailed roles of Gm26917 in macrophages, expression levels of cytokines, chemokines, chemokine receptors and phagocytosis-related genes were measured by quantitative real-time reverse-transcription PCR (qRT-PCR). We found that expression of the proinflammatory cytokines interleukin-1β (IL-1β), IL-6, IL-12 and tumor necrosis factor-α (TNF-α) was dramatically reduced, and expression of the anti-inflammatory cytokine IL-10 was increased before and after LPS treatment in the Gm26917-silenced group (Figure 3C). Expression levels of chemokines (Ccl2, Ccl3, Ccl4, Ccl24, Cxcl2, and Cxcl10) (Figure 3D), phagocytosis-related genes (Axl, Gpnmb, Merkt, Macro, Trem2, CD36) (Figure 3E) and chemokine receptors (Ccr1, CD74 and Cx3cr1) (Figure 3F) were all lower in the Gm26917 silencing group in peritoneal macrophages before and after LPS treatment. In addition, the expression levels of Cxcl3, Ccr2, Sell, CD81, and CD5L were also detected, and they showed similar patterns (Supplementary Figure S3).

We further used flow cytometry to detect the differentiation of macrophages in the control and Gm26917 silencing groups before and after LPS treatment. Consistent with the qRT–PCR results, we found that the percentage of proinflammatory macrophages (M1 macrophages) marked by Ly6chigh was significantly reduced in the Gm26917 knockdown group. LPS treatment promotes M1 macrophage polarization. After LPS treatment, the population of Ly6Chigh was also decreased in Gm26917-silenced cells (Figure 3G, upper panel).

We also tested whether the percentage of alternatively activated macrophages (M2 macrophages) marked by CD206 was increased compared control group. After LPS treatment, the population of CD206-positive macrophages was increased (Figure 3G, lower panel). In summary, we found that knocking down Gm26917 facilitated macrophage polarization from the proinflammatory M1 phenotype to the alternatively activated M2 phenotype.

### Gm26917 interacts with ANXA1

**FIGURE 4:**
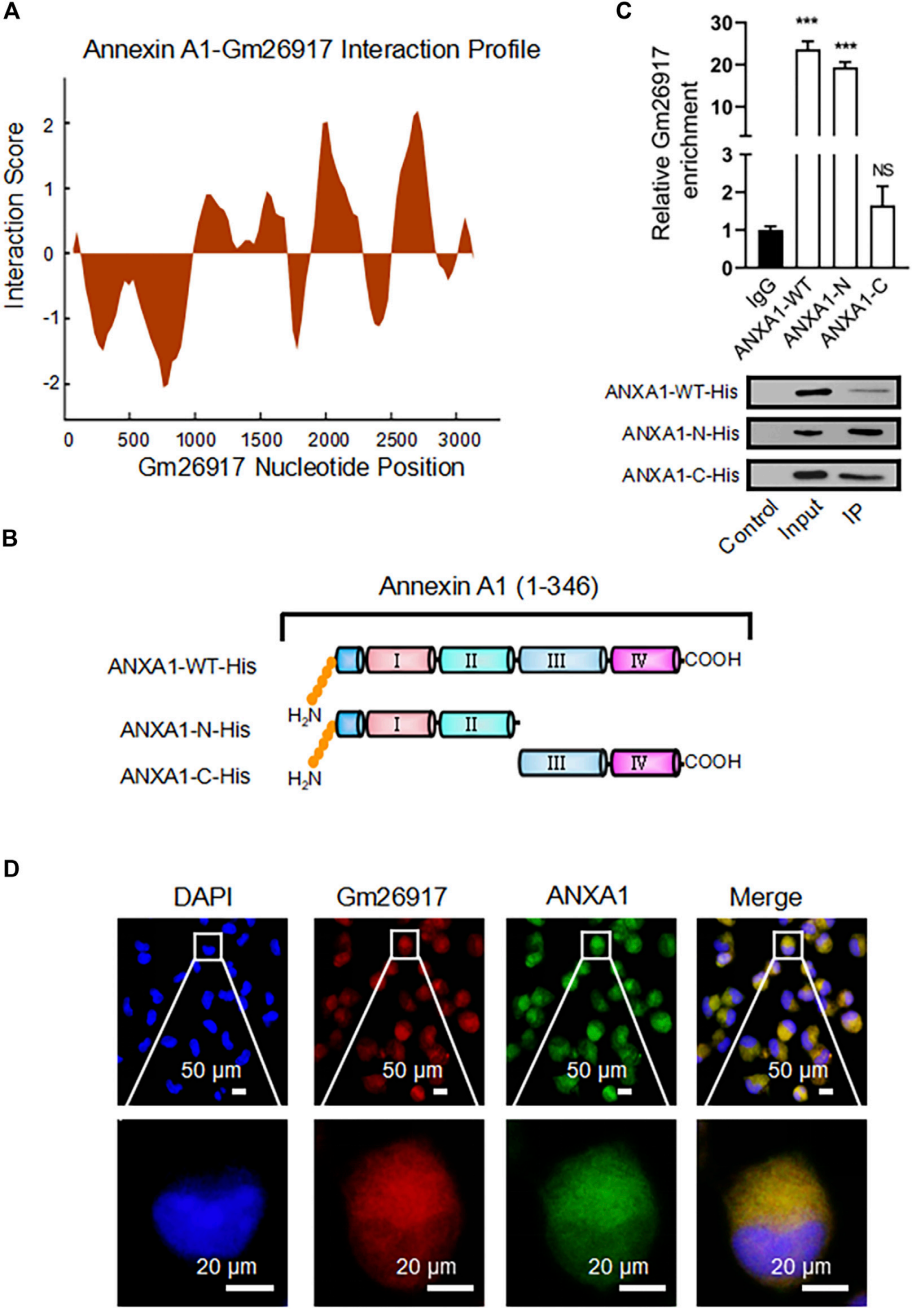
Gm26917 colocalizes with ANXA1 in the cytoplasm of macrophages. **(A)** Prediction of the interaction propensity between the RNA binding protein ANXA1 and full-length lncRNA Gm26917 by catRAPID analysis. **(B)** Diagram of WT, N-terminal and C-terminal His-tagged ANXA1 truncation mutants. **(C)** The N-terminus of ANXA1 binds to Gm26917. RNA immunoprecipitation (RIP) was performed to determine the enrichment of Gm26917 on His-tagged ANXA1 proteins in macrophages using an anti-His antibody. Western blot analysis of His-tagged ANXA1 proteins. **(D)** Gm26917 colocalized with endogenous ANXA1 in the cytoplasm of macrophages. Peritoneal macrophages were costained with Cy3-labeled anti-Gm26917 probe (red) and anti-ANXA1 antibody (green). DAPI was used to stain nuclei (blue). Data represent the mean ± SEM of three independent experiments. ^*^ *p* < 0.05, ^**^ *p* < 0.01, ^***^ *p* < 0.001.

The interaction of lncRNAs with RNA-binding proteins (RBPs) is important for their function. To identify the interaction partner of Gm26917, we first used catRAPID (http://service.tartaglialab. com/page/catrapid group) to identify putative RNA-binding proteins specific to Gm26917. CatRAPID revealed 48 proteins interacting with Gm26917 (Supplementary Table S4). CatRAPID predicted a high binding propensity between base pairs (bp) 1901 and 2,951 of Gm26917 and nearly the N-terminus of ANXA1 (Figure 4A, Supplementary Figure S4A). Interestingly, we also found two peaks in the expression of Gm26917 based on the Integrative Genomics Viewer (IGV) map of RNA-Seq reads (Supplementary Figure S4B).

To verify the prediction result, WT, N-terminal and C-terminal truncated mutants of His-tagged ANXA1 were generated (Figure 4B). Then, RNA immunoprecipitation was performed to examine whether Gm26917 interacted with ANXA1. Cell lysate was immunoprecipitated (IP) with anti-His antibody. Immunoprecipitated RNA fragments were reverse transcribed and detected by qRT–PCR using Gm26917-specific primers. We found that compared with the IgG control, the WT ANXA1 and N-terminal truncation pulled down approximately 20-fold more ANXA1, while there was no obvious enrichment with the ANXA1 C-terminal truncation (Figure 4C, upper panel). Therefore, consistent with the catRAPID prediction results, ANXA1 could interact with Gm26917 *via* its N-terminus. Protein levels of different ANXA1 proteins were examined by Western blot analysis (Figure 4C, lower panel). Moreover, Gm26917 and ANXA1 immunostaining showed that Gm26917 was clearly colocalized with ANXA1 in the cytoplasm of macrophages (Figure 4D). Taken together, these data suggests that ANXA1 interacts with Gm26917 in macrophages *via* its N-terminus.

### Gm26917 promotes ANXA1 ubiquitination

**FIGURE 5:**
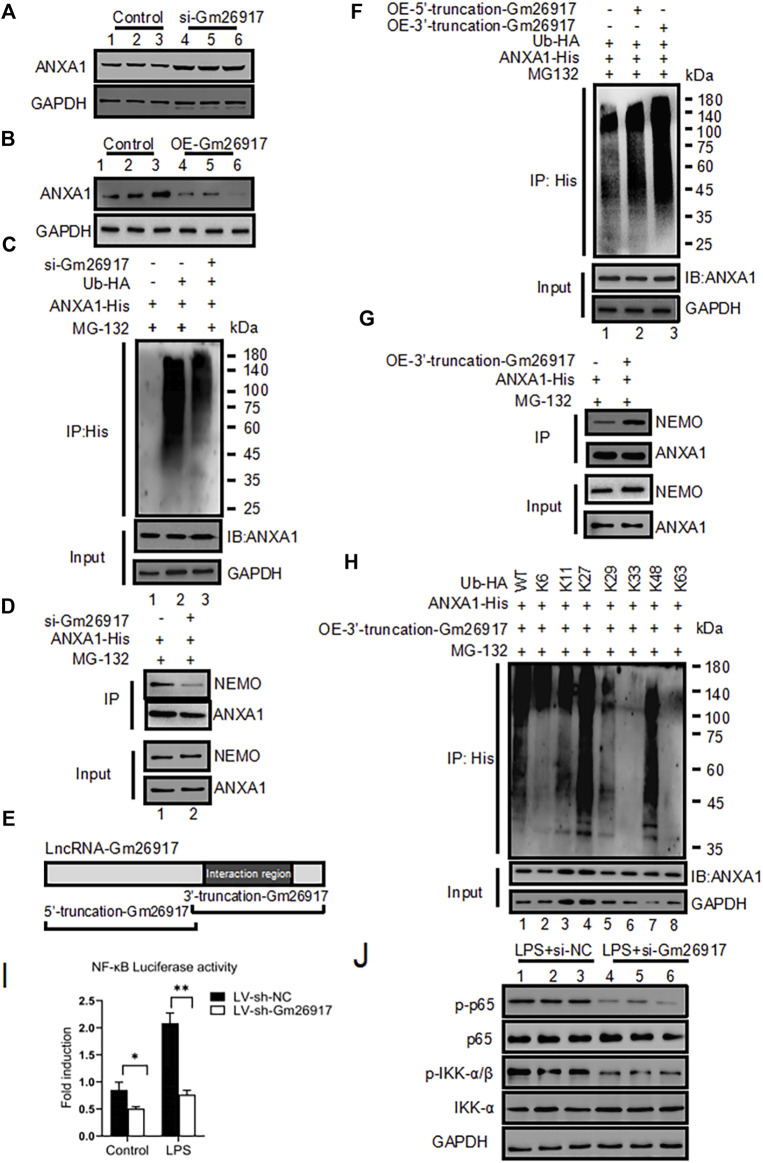
Gm26917 promotes ANXA1 ubiquitination. **(A)** Knockdown of Gm26917 increased the amount of steady-state ANXA1 protein. NIH 3T3 cells were transfected with control siRNA (scrambled, lane 1–3) or siRNA targeting Gm26917 (si-Gm26917, lane 4–6). ANXA1 expression in the control and si-Gm26917 groups was examined by Western blotting. GAPDH served as a loading control. **(B)** Overexpression of Gm26917 decreased the steady-state ANXA1 protein level. NIH 3T3 cells were transfected with control vector (lane 1–3), overexpression Gm26917 (lane 4–6). ANXA1 expression in the control and Gm26917 overexpression groups was examined by Western blotting. GAPDH served as a loading control. **(C)** Depletion of Gm26917 greatly reduced the ubiquitination of ANXA1. NIH 3T3 cells expressed ANXA1-His with control siRNA (scrambled, lane 2) or siRNA targeting Gm26917 (si-Gm26917, lane 3). Lysates were immunoprecipitated (IP) with anti-His antibodies followed by detection with anti-HA antibodies by Western blotting. The input protein ANXA1-His and loading control (GAPDH) are presented in the lower panels. **(D)** Knockdown of Gm26917 reduced the binding between ANXA1 and NEMO. **(E)** Schematic diagram of the 5′- truncation and 3′- truncation of Gm26917. **(F)** The 3′- truncation of Gm26917 promote ANXA1 ubiquitination. 293T cells were transfected with ANXA1-His, control vector (lane 1), Gm26917 5′- truncation (lane 2) or Gm26917 3′- truncation (lane 3). Lysates were immunoprecipitated (IP) with anti-His antibodies followed by detection with anti-HA antibodies by Western blotting. The input protein ANXA1-His and loading control GAPDH are presented in the lower panels. **(G)** Overexpression of the 3′-truncation of Gm26917 increased the interaction between ANXA1 and NEMO. **(H)** HEK293T cells were transiently transfected with His-tagged ANXA1 and HA-tagged Ub (mutants) vectors as indicated for 48 h. Then, polyubiquitination of ANXA1 was examined by Western blotting using an anti-HA antibody. The input protein ANXA1-His and loading control GAPDH are presented in the lower panels. **(I,J)** Gm26917 silencing inhibited NF-κB activity. **(I)** NF-κB luciferase activity was determined. Firefly luciferase activity was normalized to Renilla luciferase activity. The results are expressed as relative fold changes in luciferase activity. **(J)** Representative western blot of the effect of Gm26917 silencing on phosphorylated p65 and IKKβ in NIH 3T3 cells. Data represent the mean ± SEM of three independent experiments. ^*^ *p* < 0.05, ^**^ *p* < 0.01, ^***^ *p* < 0.001.

To further explore the relationship between Gm26917 and ANXA1, we first tested the expression of ANXA1 after Gm26917 knockdown. We found that Gm26917 silencing significantly increased ANXA1 protein expression (Figure 5A, compare lanes 4-6 with lanes 1–3), while did not change its mRNA level (Supplementary Figure S5A). In contrast, Gm26917 overexpression reduced ANXA1 protein expression levels (Figure 5B, compare lanes 4-6 with lanes 1–3). These results indicated that Gm26917 could regulate ANXA1 protein stability. Many studies have shown that lncRNAs may affect protein posttranslational modifications, such as ubiquitination. Therefore, Gm26917 could also regulate ANXA1 stability by ubiquitination.

To examine the roles of Gm26917 in ANXA1 ubiquitination, His-ANXA1 and HA-tagged ubiquitin were cotransfected into NIH 3T3 cells with the control or Gm26917 silencing. These cells were also exposed to DMSO (control) or MG132. The cell lysates were immunoprecipitated using a His antibody and immunoblotted with HA antibody. The results showed that Gm26917 knockdown using si-Gm26917 significantly reduced the ubiquitination of ANXA1 (Figure 5C, upper panel, compare lane 3 with lane 2). More interestingly, decreased ubiquitination of ANXA1 reduced its binding between nuclear factor-kappa B essential modulator (NEMO) (Figure 5D, upper panel, compare lane 2 with lane 1). Additionally, we found that Gm26917 overexpression increased the ubiquitination of ANXA1 (Supplementary Figure S5B).

The online RNA-protein binding prediction site catRAPID has predicted that the interaction region was mainly located at base pairs (bp) 1901 and 2,951 of Gm26917 (Figure 4A), which suggested that the main functional area of Gm26917 might be located in the corresponding sequence. To test this hypothesis, we constructed the corresponding Gm26917 5′-truncation expression plasmid and 3′-truncation expression plasmid to explore the main functional region of Gm26917 (Figure 5E). As shown in Figure 5F, compared with the vector control cells, overexpression of the Gm26917 3′-truncation dramatically enhanced ANXA1 ubiquitination (Figure 5F, compare lane 3 compared with lane 1), whereas Gm26917 5′-truncation had no obvious effect (Figure 5F, lane 2 compared with lane 1). The interaction between ANXA1 and NEMO was also increased after ANXA1 ubiquitination (Figure 5G, upper panel, compare lane 2 with lane 1).

To further determine the type of ubiquitin on ANXA1, HEK293T cells were transfected with plasmids expressing mutant forms of ubiquitin that were specifically ubiquitinated *via* the K6, K11, K27, K29, K33, K48, or K63 linkages. We found that the presence of Gm26917 resulted in significantly less K6-, K11-, K29-, K33- and K63-linked ubiquitin (Figure 5H, lanes 2, 3, 5, 6 and 8), but no significant change was found in K27- and K48-linked ubiquitin (Figure 5H, lanes 4 and 7). This finding indicated that Gm26917 affected K6, K11, K29, K33, and K63 branched ubiquitin chain formation, while K27 and K48 branched ubiquitin chains were not affected.

### Knockdown of Gm26917 suppresses the NF-κB signaling pathway

Many studies have shown that the TLR4/NF-κB signaling pathway is critical in inflammatory responses in LPS-activated cells. To further examine the roles of Gm26917 in the NF-κB signaling pathway, an NF-κB luciferase reporter was transfected into NIH 3T3 cells with the control or lentivirus-shRNA-Gm26917 with or without LPS treatment. We found that exogenous silencing of Gm26917 inhibited NF-κB activity before and after LPS treatment (Figure 5I). Western blot results also showed that knocking down Gm26917 decreased the phosphorylation of p65 and IKK-α upon LPS treatment (Figure 5J). Further study found that ANXA1 silencing in Gm26917 knockdown macrophages decreased the expression of pro-inflammatory cytokines (Supplementary Figure S5C) indicating the important role of ANXA1 in Gm26917 modulated TLR4/NF-κB signaling pathway. Moreover, western blot analysis also showed that LV-sh-Gm26917 pretreatment significantly suppressed p65 phosphorylation levels in the livers of LPS-induced ALI mice compared with the LV-sh-NC-treated group (Supplementary Figures S6A,B). Therefore, we concluded that Gm26917 served as an activator of the NF-κB signaling pathway.

### FOXM1 regulates Gm26917 expression

**FIGURE 6:**
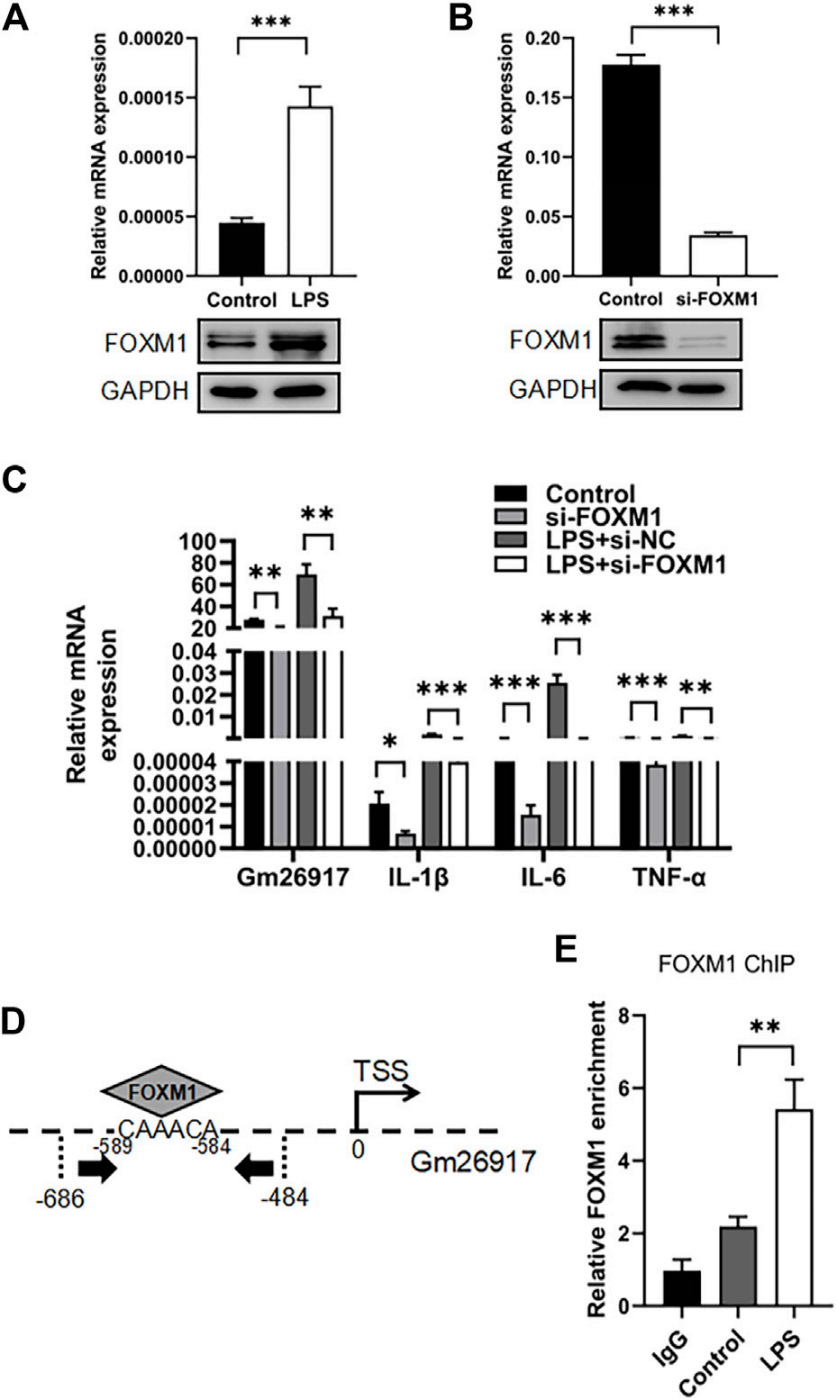
FOXM1 regulates Gm26917 expression. **(A)** LPS induced FOXM1 expression. Relative mRNA (upper panel) and protein (lower panel) expression levels of FOXM1 in LPS-treated peritoneal macrophages were examined by qRT–PCR and Western blotting. GAPDH served as a loading control. **(B)** The siRNA knockdown efficiency of FOXM1 mRNA and protein. GAPDH served as a loading control. **(C)** FOXM1 silencing decreased the expression of Gm26917 and inflammatory cytokines. The expression levels of Gm26917, IL-1β, IL-6 and TNF-α in the control and si-FOXM1 groups before and after LPS treatment in RAW264.7 cells were measured by qRT–PCR. **(D)** Schematic diagram of the upstream promoter regions of lncRNA-Gm26917. Predicted FoxM1 binding sites are shown (-589 to -584) in the Gm26917 promoter region. **(E)** FOXM1 bound to the promoter region of Gm26917. ChIP-qPCR was performed to analyze the binding of FOXM1 to the promoter of Gm26917 in control and LPS-treated RAW264.7 cells; IgG served as a negative control. Data represent the mean ± SEM of three independent experiments. ^*^ *p* < 0.05, ^**^ *p* < 0.01, ^***^ *p* < 0.001.

Gm26917 has been reported to be regulated by the transcription factor forkhead box M1 (FOXM1) in muscle satellite cells. We then test the relationship between Gm26917 and FOXM1 in macrophages. RT–qPCR revealed a 3-fold increase in FOXM1 mRNA expression after LPS challenge (Figure 6A, upper panel), and the expression of FOXM1 protein was also detected by western blot (Figure 6A, lower panel).

Next, the detail function of FOXM1 in macrophages was investigated using RAW264.7 cell line. Three FOXM1 siRNAs were generated and tested, and we found that compared with the control group, the knockdown efficiency of FOXM1 reached 80% (Figure 6B, upper panel). Western blot analysis confirmed the knockdown efficiency (Figure 6B, lower panel). qRT–PCR results showed that knockdown of FOXM1 resulted in a significant downregulation of Gm26917 (Figure 6C, left panel). Moreover, FOXM1 silencing also decreased the levels of IL-1β, IL-6 and TNF-α in RAW264.7 cells before and after LPS treatment (Figure 6C, right panel).

FOXM1-binding site was identified in the upstream regions of Gm26917 (Figure 6D). Chromatin immunoprecipitation-qPCR (ChIP-qPCR) results showed that FOXM1 bound the promoter region of Gm26917, and LPS treatment dramatically increased this binding (Figure 6E). These data have demonstrated that FOXM1 directly regulates the expression of Gm26917 in macrophages.

## Discussion

**FIGURE 7:**
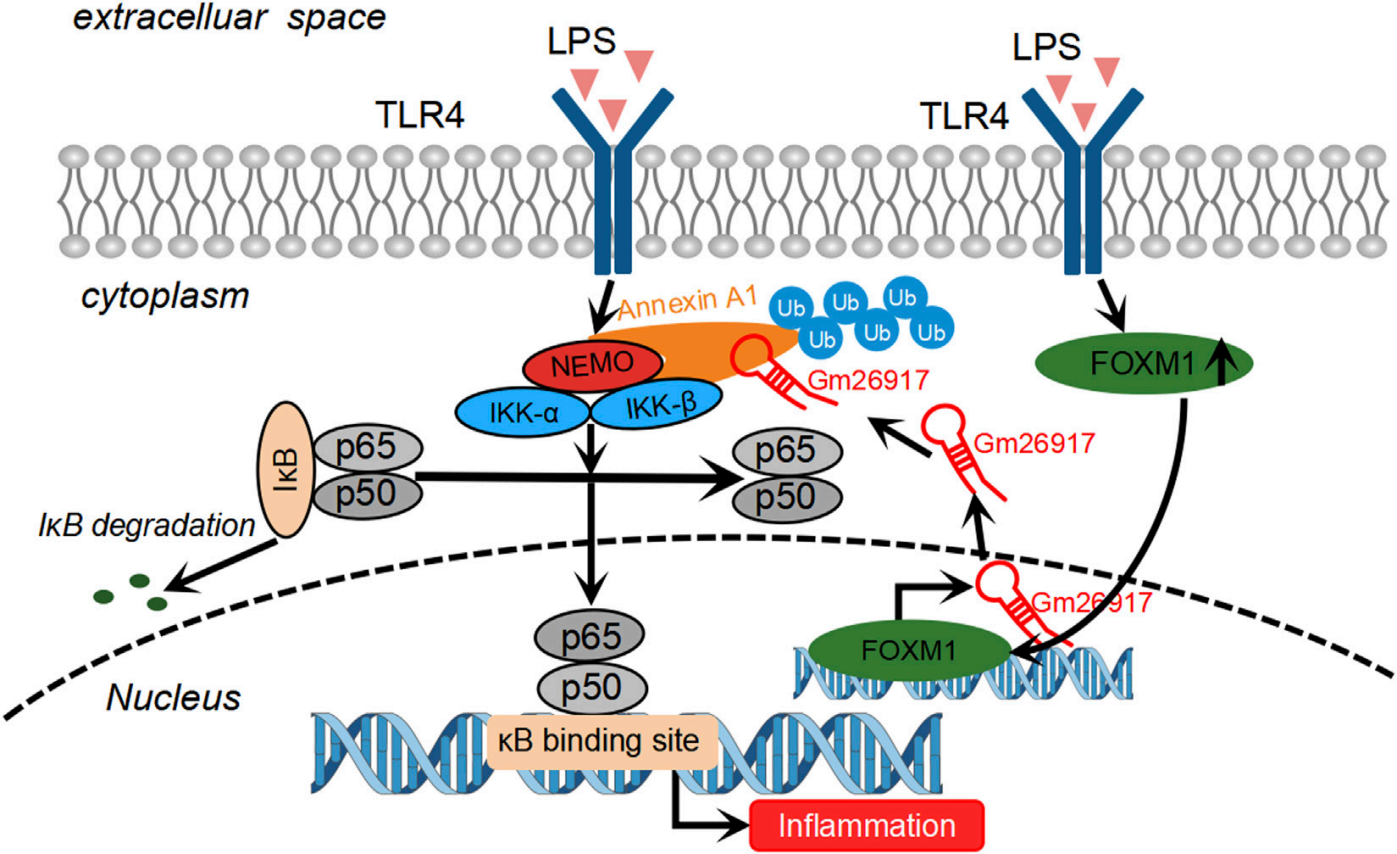
Schematic of the molecular mechanisms of Gm26917 roles in LPS-induced inflammation in macrophages. LPS challenge induces the expression of FOXM1, a transcriptional activator. FOXM1 binds to the promoter region of Gm26917 and alters the expression of Gm26917 after LPS treatment. Gm26917 specifically promotes K6-, K11-, K27-, K33- and K63-linked polyubiquitylation of ANXA1, which can enhance its interaction with NEMO and eventually activate the NF-κB signaling pathway. In this way, Gm26917 can regulate macrophage immune responses in LPS-induced acute liver injury.

As a key component of the innate immune system, macrophages play critical roles in the initiation, progression and resolution of ALI (Shan and Ju, 2020). In this study, we identified that lncRNA Gm26917 is highly expressed in activated liver macrophages. Gm26917 siliencing attenuates liver injury in LPS-induced ALI. We also found that Gm26917 could regulate NF-κB activity by modulating ANXA1 ubiquitination. Ubiquitylated ANXA1 showed superior binding to NEMO. Increased expression of Gm26917 in LPS-induced inflammation was regulated by the transcription factor FOXM1 (Figure 7). To the best of our knowledge, this is the first study to comprehensively analyze the detailed mechanistic role of Gm26917 in ALI.

Liver dysfunction is an early sign of sepsis. In recent years, several lncRNAs have been reported to be involved in septic liver injury (Zhang and Niu, 2019). For example, the lncRNA NEAT1 was up-regulated in patients with septic liver injury. Mechanistic studies have found that NEAT1 could interact with Let-7a, target TLR4, and ultimately regulate the inflammatory response in septic liver injury. Another study showed that lncRNA CRNDE was down-regulated in the CLP model of sepsis in rat. LncRNA CRNDE also acted as an endogenous competing RNA to adsorb miR-126–5p, promoting the expression of BCL2L2, and finally could attenuate septic liver injury by inhibiting hepatocyte apoptosis (Li Y. et al., 2020). In this study, we found that the Gm26917 expression level was notably upregulated in hepatic macrophages upon LPS stimulation. Knockdown of Gm26917 decreased hepatic macrophage number and relieved liver injury in LPS-induced acute liver injury (ALI).

As critical components of the innate immune system, hepatic macrophages were a remarkably heterogeneous population of immune cells that hold a central position in the initiation, progression and resolution of acute liver injury (Tacke and Zimmermann, 2014). In the past decade, many studies have shown that macrophages, which were immune cells that recognized the “plasticity” of the microenvironment, could be epigenetically programmed by signals from the tissue environment. Macrophages could be polarized into two subsets with diverse functions by specific microenvironmental stimuli and signals. The first subset consists of classically activated macrophages (M1), the key effector functions of which were bacterial clearance, antiviral activity and release of proinflammatory cytokines (such as TNF-α, IL-1β, IL-12, ROS), while the second subset comprises alternatively activated macrophages (M2) that participate in tissue remodeling and secretion of immune-modulatory mediators (such as IL-10, TGF-β, IL-4, IL-13) (Murray and Wynn, 2011). Our results showed that Gm26917 silencing globally suppressed many inflammatory pathways in macrophages. Knockdown of Gm26917 alleviated the inflammation, decreased the phagocytic and chemotactic ability of macrophages. We also demonstrated that Gm26917 silencing substantially switched macrophage polarization from the M1 to the M2 phenotype under LPS treatment.

To explore the underlying biological mechanism involved in the inflammatory immune responses mediated by Gm26917, we used catRAPID omics (Agostini et al., 2013), a server for large-scale calculations of protein-RNA interactions to predict RNA binding proteins of Gm26917. Then, RNA immunoprecipitation was utilized to verify that the ANXA1 protein bound to Gm26917 *via* its N-terminus. Fluorescence *in situ* hybridization results also showed that Gm26917 colocalized with ANXA1 in the cytoplasm of cultured macrophages. ANXA1 has been shown to be regulated by glucocorticoids, implicated in many cellular processes, including inflammatory responses, antiproliferative and proapoptotic processes, and demonstrated to regulate differentiation (Flower and Rothwell, 1994; Perretti and Gavins, 2003; Lim and Pervaiz, 2007; Shao et al., 2019). ANXA1 could be expressed by many immune cells, such as monocytes, macrophages, mast cells, neutrophils, eosinophils, epithelial cells and T cells (Costa et al., 2018). In LPS-treated macrophages, ANXA1 induced the release of IL-10 in a dose- and time-dependent manner (Minghetti et al., 1999; Parente and Solito, 2004). Additionally, another study examined ANXA1 in breast cancer found that ANXA1, could bind to NEMO at a high level, thereby stabilizing the IKK complex and ultimately inducing the activity of NF-κB (Bist et al., 2011). Moreover, ANXA1 could be posttranslationally modified by, for example, phosphorylation (Dorovkov and Ryazanov, 2004; Zhao et al., 2016; Raulf et al., 2018), acetylatation (Tabe et al., 2007), and ubiquitination (Hirata et al., 2010; Hirata et al., 2012).

Several studies have indicated that lncRNAs could regulate protein stability, which may involve protein modifications such as ubiquitination or phosphorylation (He et al., 2019). LncRNA could promote protein ubiquitination by recruiting E3 ubiquitin ligase to the target protein or enhancing the binding between the target protein and the E3 ligase by phosphorylation. LncRNAs could also promote the expression of specific E3 ligases and enhance ubiquitination of their target protein (Zhou et al., 2021). For example, lncRNA-Mirt2 served as a repressor of inflammatory responses by affecting the interaction between E3 ligase and TRAF6 (Du et al., 2017). LncRNA GBCDRlnc1 could interact with phosphoglycerate kinase 1 (PGK1) protein and regulate its stability by inhibiting its ubiquitination (Cai et al., 2019). LINC00673 was able to strengthen the interaction between PTPN11 and PRPF19 (an E3 ubiquitin ligase) and promoted the degradation of PTPN11 through ubiquitination (Zheng et al., 2016).

Ubiquitination was an important posttranslational modification of proteins, and its ability to regulate protein degradation, structure and activity was observed in almost all eukaryotic cells. Ubiquitinated ANXA1 in nuclei might be involved in the DNA damage response (Hirata et al., 2010). As previously reported, ubiquitination of ANXA1 required UbcH2A (Rad6 homolog) and a lysate containing E3 ubiquitin ligase. However, lysate pretreated with anti-Rad18 antibody did not catalyze ANXA1 ubiquitination, so Rad18 was most likely serves as the E3 ligase for ubiquitination of ANXA1 (Hirata et al., 2010). Another study found that ubiquitin-protein ligase E3A (UBE3A), also known as E6AP ubiquitin-protein ligase (E6AP), was the prototype of a family of ubiquitin ligases called HECT domain ubiquitin ligases; UBE3A interacted with the ANXA1 protein and mediated its ubiquitin-dependent degradation (Shimoji et al., 2009). In sum, the E3 ubiquitin ligase of ANXA1 is still unclear. In this study, we found that Gm26917 regulated the protein stability of ANXA1. Gm26917 silencing reduced the ubiquitination of ANXA1 and the interaction between ANXA1 and NEMO. The 3′-truncation of Gm26917 was responsible for the increased ubiquitination of ANXA1. Forced expression of the 3′-truncation of Gm26917 increased the interaction between ANXA1 and NEMO. We also demonstrated that Gm26917 could affect K6, K11, K29, K33, and K63 branched ubiquitin chain formation but not K27 and K48 branched ubiquitin chains. Gm26917 may recruit potential E3 ubiquitin ligases to ANXA1 or promote ANXA1 phosphorylation and finally enhance ANXA1 ubiquitination, but the specific E3 ubiquitin ligases are still unknown.

In a ubiquitin chain, the ubiquitin fragment could be joined by one of the lysine residues (K6, K11, K27, K29, K33, K48 and K63) or the N-terminal methionine residue, which provided the possibility of the assembly of specific types of polymerizations (Akutsu et al., 2016). Different types of ubiquitin chains were distinct intracellular signals that facilitate the diverse outcomes of ubiquitination in cells. Ubiquitination was not just a signal for protein degradation, but it has been shown to modify substrate activities and to modulate protein localization or interactions, DNA damage repair, signal transduction, endocytosis, transcriptional regulation, and cell cycle progression (Tracz and Bialek, 2021). Gm26917 could affect the biological roles of ANXA1 by promoting the formation of different ubiquitination chains on ANXA1. Further investigation of the detailed mechanism of ANXA1 ubiquitination could facilitate understanding of the function of ANXA1.

Taken together, we conclude that Gm26917, which was a LPS-induced lncRNA in the liver, specifically promoted the ubiquitination of ANXA1 and thus aggravated macrophage inflammatory responses after TLR4 activation (Figure 7). These findings may provide new opportunities for further investigations of Gm26917 as a new therapeutic target for the treatment of inflammatory liver diseases.

## Data Availability

The datasets presented in this study can be found in online repositories. The names of the repository/repositories and accession number(s) can be found below: NCBI under accession number GSE185716.
