# Supplementary material for: LncRNA Gm26917 regulates inflammatory response in macrophages by enhancing Annexin A1 ubiquitination in LPS-induced acute liver injury
Source: Front Pharmacol. 2022 Nov 1;13:975250. doi: 10.3389/fphar.2022.975250 (PMC9663662; doi:10.3389/fphar.2022.975250)
Supplement: Supplementary file 3 [file Table1.DOCX]

**Supplementary Table S1.** Quantitative real-time RT-PCR primers

| gene | Forward primer sequence | Reverse primer sequence |
| --- | --- | --- |
| Gm26917 (qRT-PCR) | GCAATTATTCCCCATGAACG | GGCCTCACTAAACCATCCAA |
| Gm26917  (ChIP) | CGGTATGGGTGACTTTGG | CACTCTGACCCGAAATCC |
| IL-1β | CTGAACTCAACTGTGAAATGC | TGATGTGCTGCTGCGAGA |
| IL-6 | ACACATGTTCTCTGGGAAATCGT | AAGTGCATCATCGTTGTTCATACA |
| TNF-α | ACTGGCAGAAGAGGCACTC | CTGGCACCACTAGTTGGTTG |
| IL-12α | CTGTGCCTTGGTAGCATCTATG | GCAGAGTCTCGCCATTATGATTC |
| IL-12β | TGGTTTGCCATCGTTTTGCTG | ACAGGTGAGGTTCACTGTTTCT |
| IL-10 | TGGCCCAGAAATCAAGGAGC | CAGCAGACTCAATACACACT |
| Ccl2 | CCAGCAAGATGATCCCAATG | TACGGGTCAACTTCACATTC |
| Ccl3 | GATTCCACGCCAATTCATCG | AGGCATTCAGTTCCAGGTCA |
| Ccl4 | TTTCTCTTACACCTCCCGGC | AGCTGCTCAGTTCAACTCCA |
| Ccl24 | CTCCTTCTCCTGGTAGCCTG | ATGGCCCTTCTTGGTGATGA |
| Cxcl2 | ACCAACCACCAGGCTACA | TCAGGGTCAAGGCAAACT |
| Cxcl3 | CCACCAACCACCAGGCTACA | GAGGCAAACTTCTTGACCATCC |
| Cxcl10 | TCATCCCTGCGAGCCTATCC | TGCGTGGCTTCACTCCAGTT |
| Axl | ATGGCCGACATTGCCAGTG | CGGTAGTAATCCCCGTTGTAGA |
| Gpnmb | CATTCCCATCTCGAAGGTGAAA | AAATGGCAGAGTCGTTGAGGA |
| Mertk | CAGGGCCTTTACCAGGGAGA | TGTGTGCTGGATGTGATCTTC |
| Macro | ACAGAGCCGATTTTGACCAAG | CAGCAGTGCAGTACCTGCC |
| Trem2 | CTGGAACCGTCACCATCACTC | CGAAACTCGATGACTCCTCGG |
| Cd36 | ATGGGCTGTGATCGGAACTG | GTCTTCCCAATAAGCATGTCTCC |
| Ccr1 | CTCATGCAGCATAGGAGGCTT | ACATGGCATCACCAAAAATCCA |
| Cd74 | CCGCCTAGACAAGCTGACC | ACAGGTTTGGCAGATTTCGGA |
| Cx3cr1 | GAGTATGACGATTCTGCTGAGG | CAGACCGAACGTGAAGACGAG |
| Cxcl3 | CCACCAACCACCAGGCTACA | GAGGCAAACTTCTTGACCATCC |
| Ccr2 | TGTGATTGACAAGCACTTAGACC | TGGAGAGATACCTTCGGAACTT |
| Cd81 | GTGGAGGGCTGCACCAAAT | GACGCAACCACAGAGCTACA |
| Sell | TACATTGCCCAAAAGCCCTTAT | CATCGTTCCATTTCCCAGAGTC |
| Cd5l | GATCGTGTTTTTCAGAGTCTCCA | TGCAGTCAACCCCTTGAATAAG |
| Gapdh | TTCACCACCATGGAGAAGGC | GGCATGGACTGTGGTCATGA |
