# Supplementary material for: LncRNA Gm26917 regulates inflammatory response in macrophages by enhancing Annexin A1 ubiquitination in LPS-induced acute liver injury
Source: Front Pharmacol. 2022 Nov 1;13:975250. doi: 10.3389/fphar.2022.975250 (PMC9663662; doi:10.3389/fphar.2022.975250)
Supplement: Supplementary file 4 [file Table5.DOCX]

## Supplementary Figures


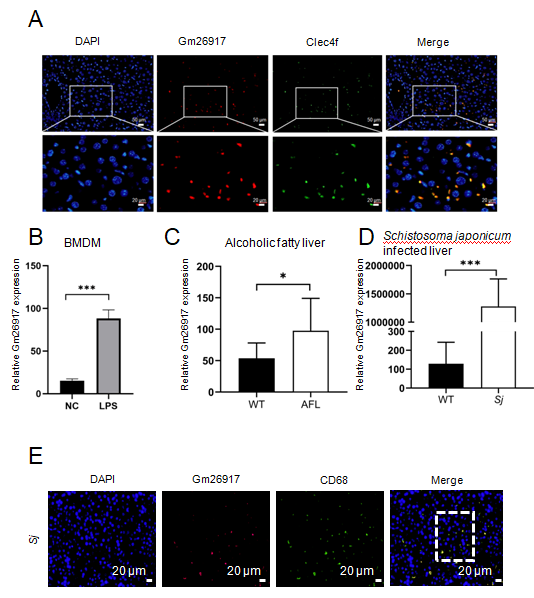


**Supplementary Figure S1. Gm26917 roles in macrophages.**

**(A)** Gm26917 colocalize with Clec4f-positive macrophages. **(B)** Gm26917 highly expressed in BMDM. **(C)** Gm26917 was induced in alcoholic fatty liver. The expression levels of Gm26917 in livers from an alcoholic fatty liver disease mouse model were quantified by qRT–PCR. **(D)** Gm26917 was induced in schistosomiasis. The expression levels of Gm26917 in liver using a mouse modelof schistosomiasis were determined by qRT–PCR. **(E)** Gm26917 localized in macrophages in these livers. For colocalization analysis, the livers were costained for Gm26917 (red) and CD68 (green, macrophage marker). Nuclei were stained with 4’,6-diamidino-2-phenylindole (DAPI) (blue). Data represent the mean±SEM of three independent experiments. ^*^*P*< 0.05, ^**^*P*< 0.01, ^***^*P*< 0.001.

**
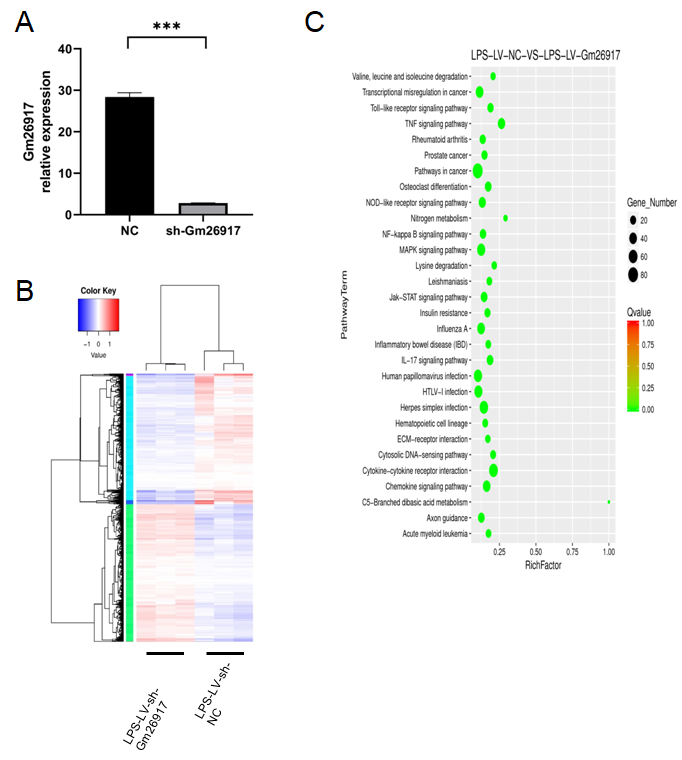
**

**Supplementary Figure S2. RNA-Seq and KEGG pathway analysis after Gm26917 knockdown in primary peritoneal macrophages.**

**(A)** Gm26917 was significantly knocked down in peritoneal macrophages. **(B)** Hierarchical clustering of all altered genes in control and sh-Gm26917-treated peritoneal macrophages, assessing three repeats. **(C)** Pathway enrichment analysis of differentially expressed genes was performed by KEGG.


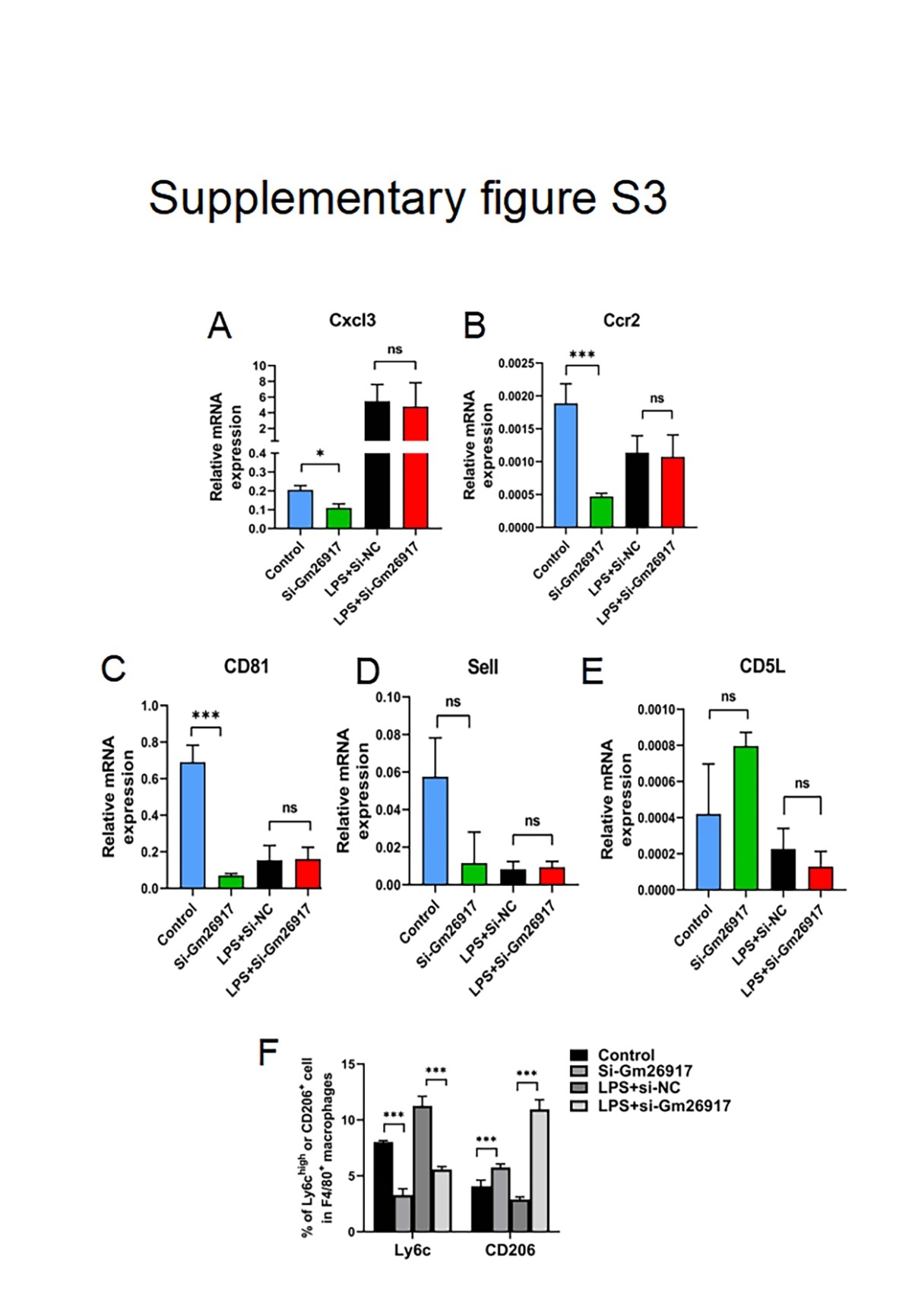


**Supplementary Figure S3.Gm26917 expression in response to different concentrations of LPS and efficiency test of si-Gm26917.** Effects of Gm26917 knockdown on the expression of Cxcl3 **(A)**, Ccr2 **(B)**, CD81 **(C)**, Sell **(D)**, and CD5L **(E)** before and after LPS treatment in cultured peritoneal macrophages, as determined by qRT–PCR. Data represent the mean±SEM of three independent experiments. ^*^*P*< 0.05, ^**^*P*< 0.01, ^***^*P*< 0.001.


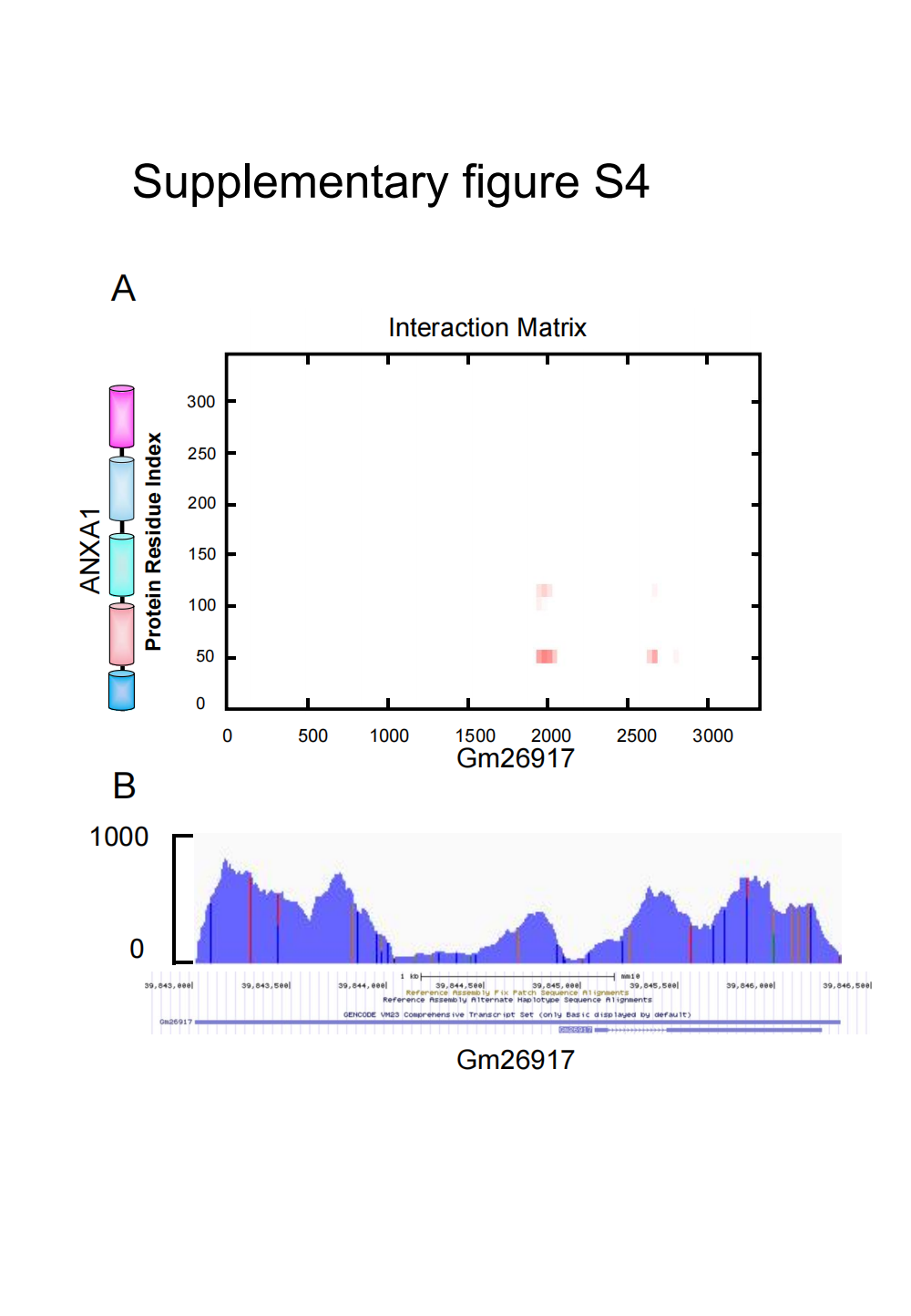


**Supplementary Figure S4. Prediction of the interaction region between Gm26917 and ANXA1. (A)** CatRAPID fragment module prediction of the interaction profile between the RNA binding protein ANXA1 and lncRNA Gm26917. **(B)** Visualization of Gm26917 RNA sequencing readsbased on the transcriptomic analysis of mouse liver shown in Figure 1A using the Integrative Genomics Viewer (IGV).

**
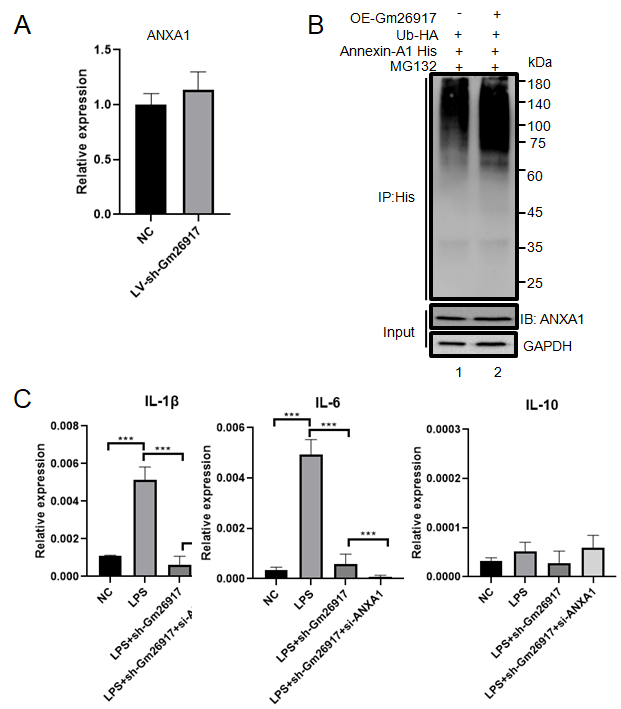
**

**Supplementary Figure S5. Gm26917 controls the protein stability and function of ANXA1.**

**(A)** mRNA expression of ANXA1 after Gm26917 silencing. **(B)** The 293T cells were transfected with Ub-HA, ANXA1-His, control vector (lane 1) or Gm26917 overexpression plasmid (lane 2). Lysates were immunoprecipitated (IP) with anti-His antibodies followed by detection with anti-HA or anti-NEMO antibodies by Western blotting. The input protein ANXA1-His and loading control (GAPDH) are presented in the lower panels. **(C)** Expression of IL-1β, IL-6 and IL-10 after ANXA1 silencing.


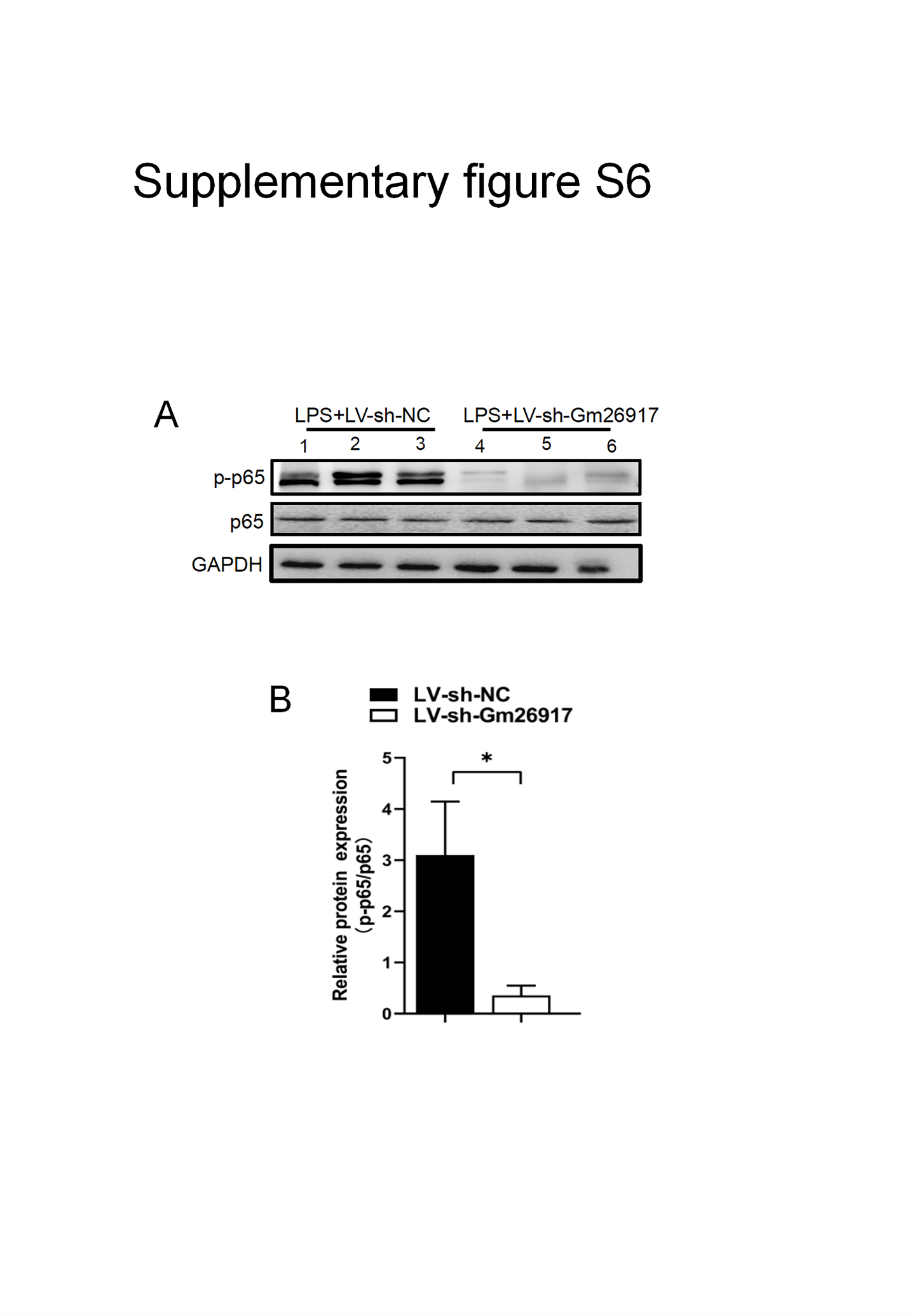


**Supplementary Figure S6. (A)** Western blot analysis of the phosphorylation of p65 in the livers of control and LPS-treated mice. **(B)** Quantification of the relative p-p65 relative expression in panel A.
