# Supplementary material for: LncRNA Gm26917 regulates inflammatory response in macrophages by enhancing Annexin A1 ubiquitination in LPS-induced acute liver injury
Source: Front Pharmacol. 2022 Nov 1;13:975250. doi: 10.3389/fphar.2022.975250 (PMC9663662; doi:10.3389/fphar.2022.975250)
Supplement: Supplementary file 5 [file Table4.DOCX]

**Supplementary Table S4.** List of proteins which interact with Gm26917 based on catRAPID Database.

| # | Protein ID | the predicted binding region of Gm26917 and protein | Z-score | Discriminative Power (%) | Interaction Strength (%) | |
| --- | --- | --- | --- | --- | --- | --- |
| 1 | ANXA1_MOUSE | lncRNA Gm26917_1605bp-1777bp | 0.85 | 96 | 100 |  |
| 2 | ANXA1_MOUSE | lncRNA Gm26917_1591bp-1777bp | 0.42 | 85 | 99 |  |
| 3 | ANXA1_MOUSE | lncRNA Gm26917_1585bp-1772bp | 0.60 | 92 | 99 |  |
| 4 | ANXA1_MOUSE | lncRNA Gm26917_1572bp-1770bp | 1.08 | 97 | 99 |  |
| 5 | ANXA1_MOUSE | lncRNA Gm26917_1613bp -1778bp | 0.32 | 81 | 98 |  |
| 6 | ANXA1_MOUSE | lncRNA Gm26917_1600bp -1777bp | 0.34 | 81 | 98 |  |
| 7 | CBX8_MOUSE | lncRNA Gm26917_1585bp -1777bp | 0.60 | 92 | 100 |  |
| 8 | CBX8_MOUSE | lncRNA Gm26917_1580bp -1772bp | 0.72 | 95 | 100 |  |
| 9 | RED1_MOUSE | lncRNA Gm26917_1656bp -1773bp | 0.04 | 63 | 91 |  |
| 10 | RED1_MOUSE | lncRNA Gm26917_1638bp -1777bp | 0.08 | 67 | 92 |  |
| 11 | RED1_MOUSE | lncRNA Gm26917_1633bp -1776bp | 0.08 | 67 | 93 |  |
| 12 | TRUB1_MOUSE | lncRNA Gm26917_1620bp -1777bp | 0.31 | 80 | 98 |  |
| 13 | TRUB1_MOUSE | lncRNA Gm26917_1600bp -1777bp | 0.37 | 84 | 98 |  |
| 14 | CRNL1_MOUSE | lncRNA Gm26917_1665bp -1772bp | 0.11 | 69 | 95 |  |
| 15 | CRNL1_MOUSE | lncRNA Gm26917_1661bp -1772bp | 0.14 | 71 | 97 |  |
| 16 | CRNL1_MOUSE | lncRNA Gm26917_1656bp -1773bp | 0.04 | 63 | 90 |  |
| 17 | CRNL1_MOUSE | lncRNA Gm26917_1652bp -1777bp | 0.22 | 76 | 96 |  |
| 18 | CBX8_MOUSE | lncRNA Gm26917_1591bp -1777bp | 0.40 | 85 | 100 |  |
| 19 | RED1_MOUSE | lncRNA Gm26917_1652bp -1777bp | 0.20 | 75 | 95 |  |
| 20 | RM01_MOUSE | lncRNA Gm26917_1620bp -1777bp | 0.28 | 79 | 98 |  |
| 21 | THOC3_MOUSE | lncRNA Gm26917_1600bp -1777bp | 0.34 | 83 | 99 |  |
| 22 | THOC3_MOUSE | lncRNA Gm26917_1591bp -1777bp | 0.38 | 84 | 99 |  |
| 23 | THOC3_MOUSE | lncRNA Gm26917_1585bp -1772bp | 0.55 | 91 | 99 |  |
| 24 | NKRF_MOUSE | lncRNA Gm26917_1638bp -1777bp | 0.04 | 63 | 91 |  |
| 25 | NKRF_MOUSE | lncRNA Gm26917_1633bp -1776bp | 0.04 | 63 | 92 |  |
| 26 | CRNL1_MOUSE | lncRNA Gm26917_1638bp -1777bp | 0.04 | 63 | 91 |  |
| 27 | CRNL1_MOUSE | lncRNA Gm26917_1633bp -1776bp | 0.05 | 63 | 92 |  |
| 28 | TRM1L_MOUSE | lncRNA Gm26917_1633bp -1776bp | 0.05 | 63 | 94 |  |
| 29 | RED1_MOUSE | lncRNA Gm26917_1661bp -1772bp | 0.08 | 67 | 96 |  |
| 30 | IF2A_MOUSE | lncRNA Gm26917_1656bp -1773bp | -0.01 | 59 | 99 |  |
| 31 | ANXA1_MOUSE | lncRNA Gm26917_1580bp -1772bp | 0.62 | 92 | 99 |  |
| 32 | PUS10_MOUSE | lncRNA Gm26917_1585bp -1772bp | 0.52 | 91 | 99 |  |
| 33 | TRUB1_MOUSE | lncRNA Gm26917_1591bp -1777bp | 0.35 | 83 | 98 |  |
| 34 | TRUB1_MOUSE | lncRNA Gm26917_1585bp -1772bp | 0.52 | 90 | 99 |  |
| 35 | IF2A_MOUSE | lncRNA Gm26917_1661bp -1772bp | 0.06 | 66 | 99 |  |
| 36 | IF2A_MOUSE | lncRNA Gm26917_1620bp -1777bp | 0.24 | 77 | 99 |  |
| 37 | QOR_MOUSE | lncRNA Gm26917_1620bp -1777bp | 0.23 | 77 | 98 |  |
| 38 | TRM1L_MOUSE | lncRNA Gm26917_1638bp -1777bp | 0.02 | 61 | 92 |  |
| 39 | TRM1L_MOUSE | lncRNA Gm26917_1620bp -1777bp | 0.24 | 77 | 96 |  |
| 40 | QKI_MOUSE | lncRNA Gm26917_1591bp -1777bp | 0.33 | 81 | 97 |  |
| 41 | QKI_MOUSE | lncRNA Gm26917_1585bp -1772bp | 0.50 | 90 | 99 |  |
| 42 | CBX8_MOUSE | lncRNA Gm26917_1572bp -1770bp | 0.94 | 97 | 100 |  |
| 43 | AUHM_MOUSE | lncRNA Gm26917_1600bp -1777bp | 0.30 | 80 | 99 |  |
| 44 | RED1_MOUSE | lncRNA Gm26917_1620bp -1777bp | 0.23 | 76 | 94 |  |
| 45 | TRUB1_MOUSE | lncRNA Gm26917_1613bp -1778bp | 0.23 | 77 | 97 |  |
| 46 | QKI_MOUSE | lncRNA Gm26917_1600bp -1777bp | 0.29 | 80 | 96 |  |
| 47 | CBX6_MOUSE | lncRNA Gm26917_1585bp -1772bp | 0.49 | 89 | 100 |  |
| 48 | PUS10_MOUSE | lncRNA Gm26917_1591bp -1777bp | 0.32 | 81 | 99 |  |
